# Deacetylation of mycothiol-derived ‘waste product’ triggers the last biosynthetic steps of lincosamide antibiotics†
†Electronic supplementary information (ESI) available: Experimental procedures, additional figures, chromatograms and NMR data. See DOI: 10.1039/c5sc03327f



**DOI:** 10.1039/c5sc03327f

**Published:** 2015-10-01

**Authors:** Zdenek Kamenik, Stanislav Kadlcik, Bojana Radojevic, Petra Jiraskova, Marek Kuzma, Radek Gazak, Lucie Najmanova, Jan Kopecky, Jiri Janata

**Affiliations:** a Institute of Microbiology ASCR , Videnska 1083 , Prague 4 , Czech Republic . Email: janata@biomed.cas.cz; b Crop Research Institute , Drnovska 507 , Prague 6 , Czech Republic

## Abstract

The immediate post-condensation steps in lincomycin biosynthesis are reminiscent of the mycothiol-dependent detoxification system of actinomycetes. This machinery provides the last proven lincomycin intermediate, a mercapturic acid derivative, which formally represents the ‘waste product’ of the detoxification process. We identified and purified new lincomycin intermediates from the culture broth of deletion mutant strains of *Streptomyces lincolnensis* and tested these compounds as substrates for proteins putatively involved in lincomycin biosynthesis. The results, based on LC-MS, in-source collision-induced dissociation mass spectrometry and NMR analysis, revealed the final steps of lincomycin biosynthesis, *i.e.* conversion of the mercapturic acid derivative to lincomycin. Most importantly, we show that deacetylation of the *N*′-acetyl-*S*-cysteine residue of the mercapturic acid derivative is required to ‘escape’ the detoxification-like system and proceed towards completion of the biosynthetic pathway. Additionally, our results, supported by l-cysteine-^13^C_3_, ^15^N incorporation experiments, give evidence that a different type of reaction catalysed by the homologous pair of pyridoxal-5′-phosphate-dependent enzymes, LmbF and CcbF, forms the branch point in the biosynthesis of lincomycin and celesticetin, two related lincosamides.

## Abstract 2

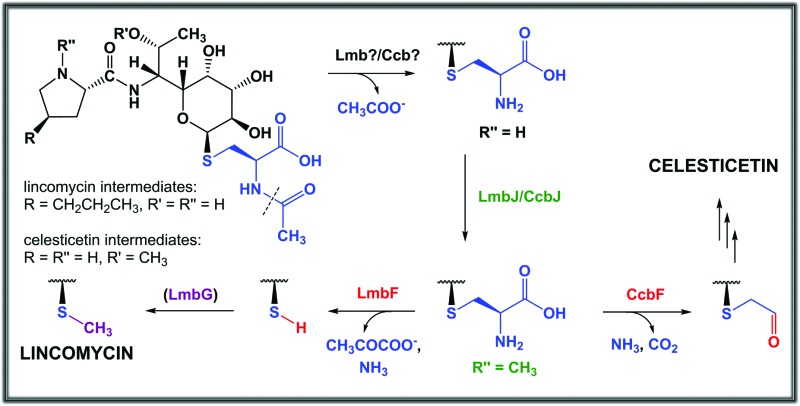
Two homologous pyridoxal 5′-phosphate-dependent enzymes LmbF and CcbF transform the deacetylated *S*-cysteinyl residue of related intermediates in the biosynthesis of lincomycin/celesticetin in different ways.

## Introduction

**Fig. 1:**
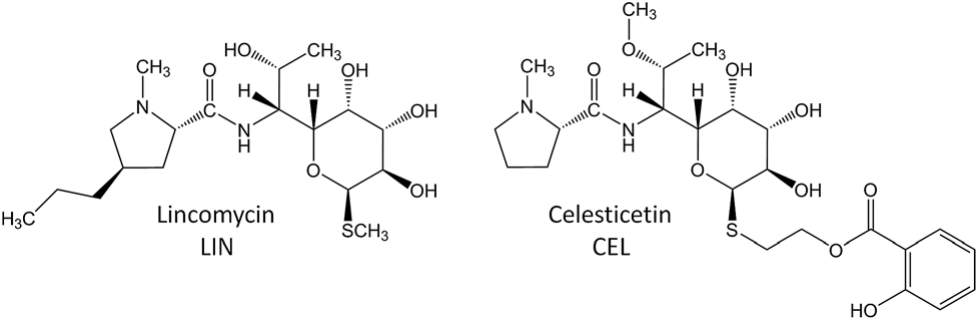
Structures of the main natural lincosamide antibiotics.

**Fig. 2:**
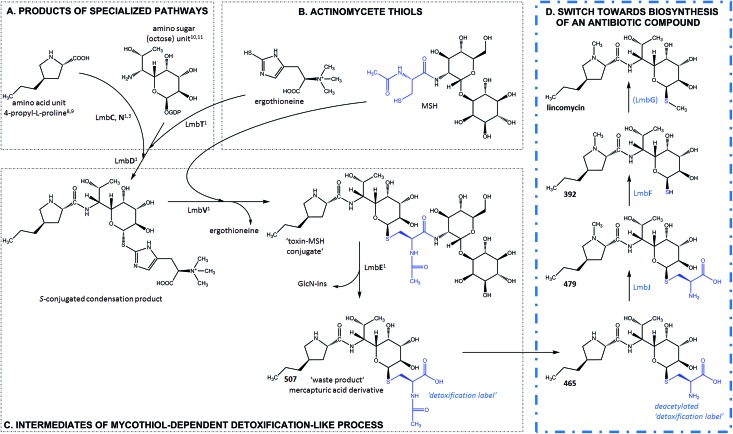
Lincomycin biosynthesis. Condensation and MSH-dependent detoxification-like process ((A–C), grey frame – evidenced previously^1,3,8–11^); switch towards biosynthesis of an antibiotic compound ((D), blue frame – evidenced in this paper). The blue structural features highlight the *N*′-acetyl-*S*-cysteine residue of MSH and its processing during lincomycin maturation. GlcN-Ins – 1-*O*-glucosamine-d-myo-inositol; MSH – mycothiol; (LmbG) – the assignment of the catalysing enzyme based on bioinformatic analysis.

Zhao *et al.* recently discovered the intriguing participation of two low-molecular-weight thiols, mycothiol (MSH) and ergothioneine, in the biosynthesis of the lincosamide antibiotic, lincomycin A (hereinafter lincomycin or LIN; Fig. 1).^1^ Such unusual involvement of these actinomycete thiols (Fig. 2B) in biosynthesis had remained undiscovered for a long time, mainly because the sulfur atom of MSH remains the only footprint of the thiols in the lincomycin structure. Ergothioneine is, however, required for condensation of the amino acid and amino octose unit to obtain the scaffold of lincomycin, whereas MSH plays its role immediately after the condensation. In the condensation process, the stand-alone adenylation domain LmbC catalyses 4-propyl-l-proline activation and transfer on the *holo*-form of the carrier protein domain of LmbN,^2,3^ whereas LmbT catalyses the formation of the octose conjugate with ergothioneine.^1^ This conjugate is then condensed with the activated 4-propyl-l-proline *via* an amide bond in a reaction catalysed by LmbD (Fig. 2A–C).^1^ Interestingly, the following post-condensation steps are at least formally reminiscent of the MSH-dependent detoxification system present in actinomycetes, which is generally used for the elimination of electrophilic toxins, including various antibiotics and their metabolites, from the cell.^4^ Following this parallel, ergothioneine is from the *S*-conjugated condensation product replaced for MSH by nucleophilic substitution in a reaction catalysed by LmbV.^1^ The resulting product corresponds to a ‘toxin’–MSH conjugate, where the lincomycin scaffold formally represents the ‘toxin’.

Subsequently, LmbE cleaves off the pseudo-disaccharide 1-*O*-glucosamine-d-myo-inositol, resulting in compound **507**, a mercapturic acid derivative characterized by the *N*′-acetyl-*S*-cysteine residue, the only remainder of MSH in its structure (Fig. 2C).^1^ The structure of **507** corresponds to the ‘waste product’ of the MSH-dependent detoxification system, where mercapturic acid derivatives are generally predetermined for transport out of the cell.^4,5^ Thus, the *N*′-acetyl-*S*-cysteinyl of the mercapturic acid derivative may be considered as a ‘detoxification label’. Interestingly, the seeming ‘waste product’ **507** is further matured to give the antibiotic product lincomycin. When comparing the structures of **507** and lincomycin, it is obvious that the *N*′-acetyl-*S*-cysteinyl ‘detoxification label’ of **507** has to be removed and that two methylations at the nitrogen and sulfur atoms have to occur to give rise to lincomycin. The *N*-methylation has been demonstrated to be catalysed by LmbJ in the biosynthesis of lincomycin and its homologue CcbJ in the biosynthesis of the related lincosamide, celesticetin (Fig. 1).^6^ Moreover, the crystal structure of the homohexamer CcbJ has been solved, and the detailed reaction mechanism has been proposed.^7^ However, the mechanism that switches the machinery from the ‘detoxification’ mode to the biosynthetic mode and how the final biosynthetic steps proceed remains elusive.

Here, we demonstrate that the acetyl group of the *N*′-acetyl-*S*-cysteinyl ‘detoxification label’ represents a locked door for further maturation of the ‘waste product’ **507** into the antibiotic lincomycin. Furthermore, we present the complete processing of the ‘detoxification label’ so that only its sulfur atom remains in the structure of lincomycin. In addition, we discuss apparent differences in the final steps of lincomycin and celesticetin biosynthesis.

## Results and discussion

### New intermediates of lincomycin biosynthesis reveal conversion of ‘waste product’ **507** to antibiotic lincomycin

**Fig. 3:**
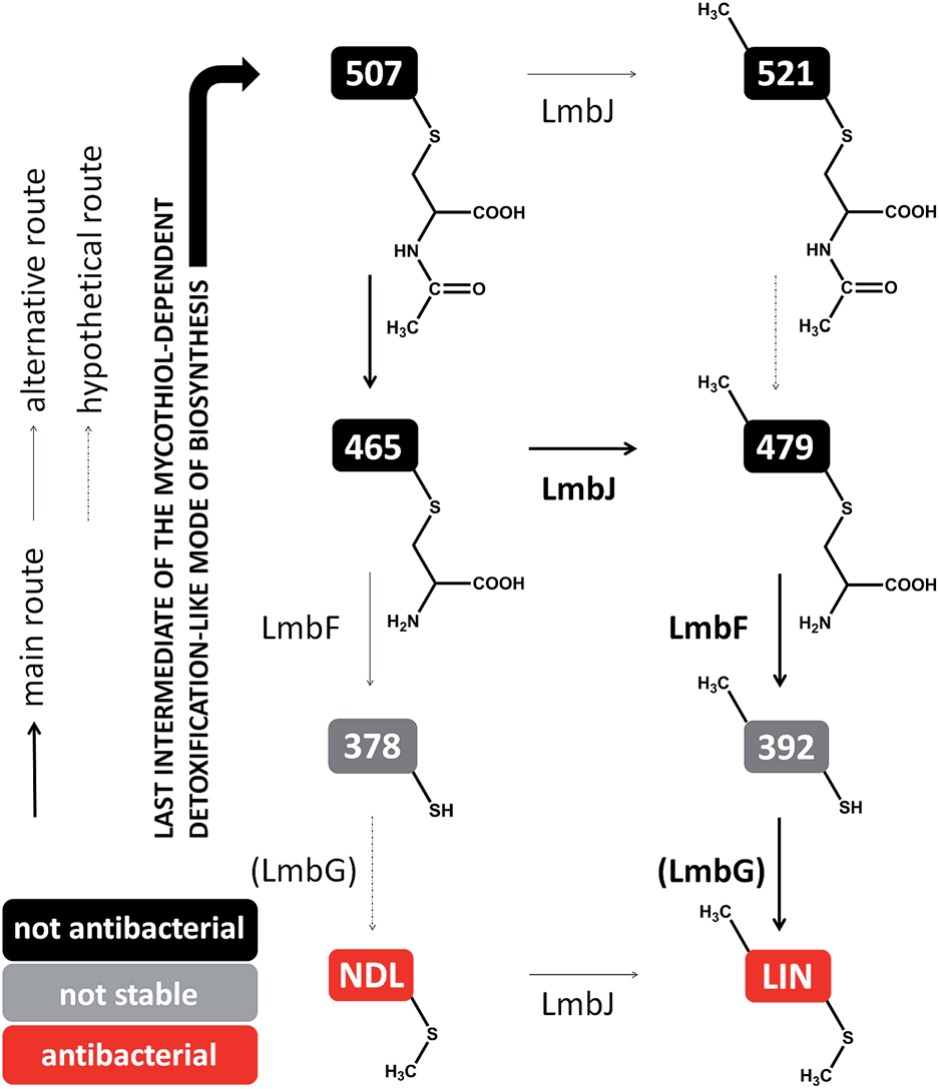
Post-condensation steps of lincomycin biosynthesis leading from **507** (exhibiting no antimicrobial activity) to the final antibiotic.

We have detected four intermediates of lincomycin biosynthesis in the culture broths of two mutant strains of the lincomycin producer, *Streptomyces lincolnensis*, with specific genes deleted (see Experimental in ESI†). Apart from the known intermediate **507** (Fig. 2C), three new hypothetical intermediates, **465**, **479** (Fig. 2D) and **521**, were found (compounds are named according to their nominal mass). The compounds mutually differ in the presence of the *N*-methyl group in their structures (present only in **479** and **521**) and in the presence of the intact *N*′-acetyl-*S*-cysteinyl ‘detoxification label’ (present only in **507** and **521**, whereas **465** and **479** have the ‘detoxification label’ deacetylated); see the graphic cartoon in Fig. 3 for the structural differences. We identified the compounds based on high resolution mass spectrometry (MS) and in-source collision-induced dissociation MS fragmentation and purified them from culture broths (Fig. S1†). The structures were further elucidated by NMR analysis (NMR data in Table S2,† ^1^H and ^13^C NMR spectra in Fig. S2†) and by incorporation experiments with l-cysteine-^13^C_3_,^15^N, which confirmed that l-cysteine is part of the molecular structure of the four intermediates (details in Fig. S3†).

The compounds were then tested *in vitro* as substrates of the recombinant proteins LmbJ and LmbF to interrogate the pathway from **507** to lincomycin (the purity and integrity can be assessed from SDS-PAGE gels in Fig. S4†). The cartoon in Fig. 3 summarizes the results presented and is discussed in more detail below. Specifically, we demonstrate that the main biosynthetic route proceeds as follows: first, compound **507**, the proven product of the LmbE reaction,^1^ is deacetylated by a yet unassigned enzyme to yield **465**, then *N*-methylated by LmbJ to yield **479**, which is subsequently converted into **392** by LmbF and finally *S*-methylated, presumably by LmbG, to give lincomycin. Additionally, we show that the relaxed substrate specificities of biosynthetic proteins enable either documented alternative or hypothetical bypasses (see Fig. 2D for the main stream; cartoon in Fig. 3 for all possible streams).

### Ornamentation by LmbJ *N*-methyltransferase is not the last biosynthetic step

**Fig. 4:**
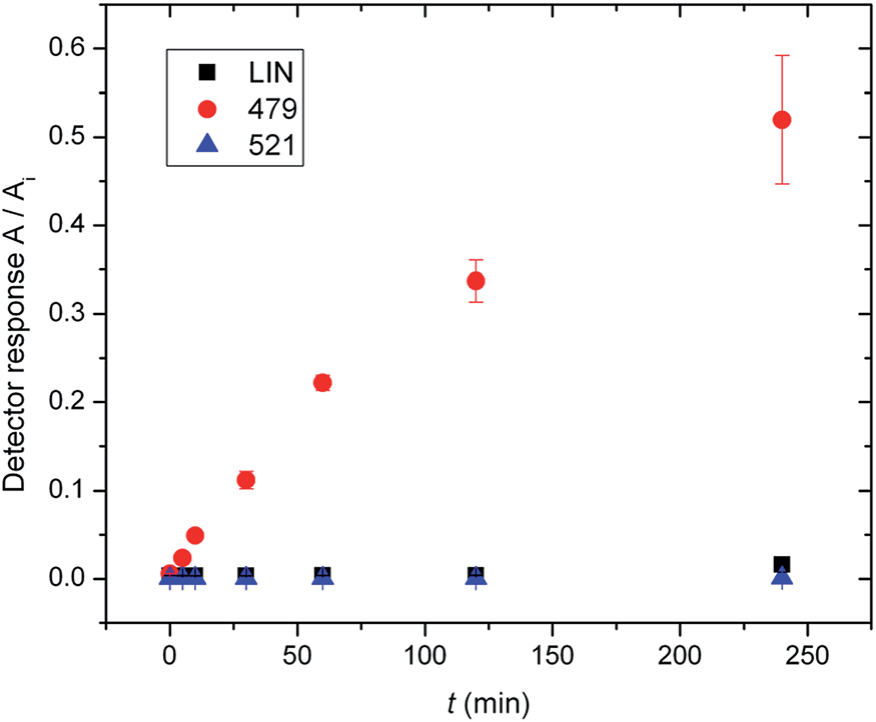
Formation of **521**, **479** and LIN products from **507**, **465** and NDL substrates, respectively, in a competitive *in vitro* assay with LmbJ. Equimolar amounts of substrates were tested simultaneously in one reaction; *n* = 3. *A* – peak area of the product, *A*_i_ – peak area of the internal standard.

In previous work by Najmanova *et al.*,^6^ it has been shown that LmbJ *N*-methylates the chemically synthesized *N*-demethyllincomycin (NDL), suggesting that *N*-methylation of the 4-propyl-l-proline moiety is the last step in lincomycin biosynthesis. However, the existence of the biosynthesized *N*-methylated intermediates **521** and **479** raises questions about whether the *N*-methylation occurs at an earlier stage. Indeed, we tested recombinant LmbJ with **507**, **465** and NDL and revealed that all of these substrates can be converted into the *N*-methylated products **521**, **479** and lincomycin, respectively (see Fig. 3 for the scheme and Fig. S5† for the LC-MS data). However, the rapid and preferential conversion of **465** but not **507** or NDL in a competitive *in vitro* assay strongly suggests that **465** is the main natural substrate of LmbJ (Fig. 4). This finding also provides an explanation of the previously observed similar kinetic parameters of the homologous *N*-methyltransferases LmbJ and CcbJ for the conversion of NDL into lincomycin:^6^ NDL is not the natural substrate for any of these two proteins. Moreover, these results confirm the broad substrate specificity of LmbJ, as predicted.^7^

### ‘Detoxification label’ is removed by LmbF only if the ‘label’ is first deacetylated

**Fig. 5:**
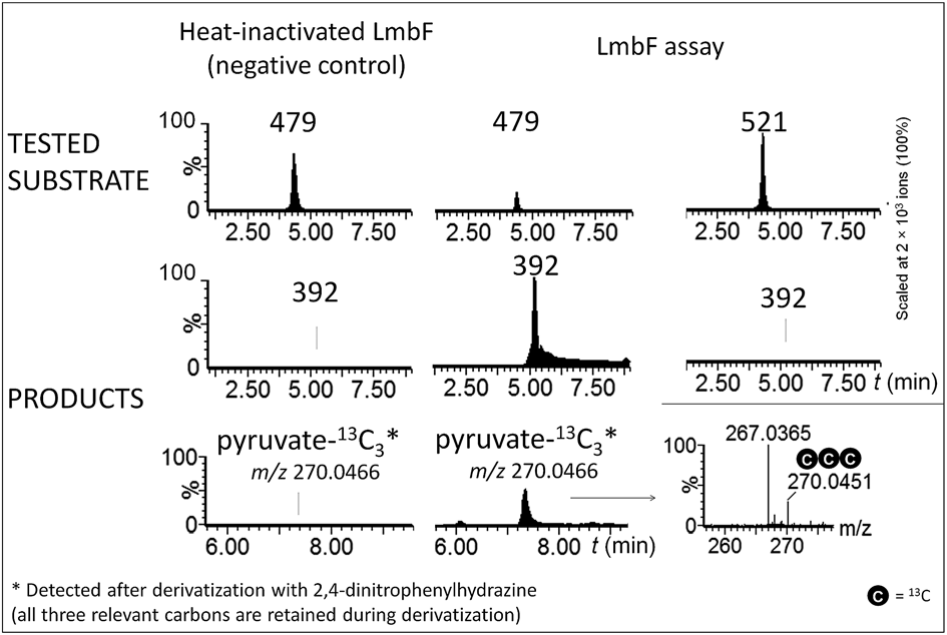
Ion-extracted LC-MS chromatograms of LmbF *in vitro* assays with **479** and **521** as substrates. Pyruvate-^13^C_3_ was detected when **479**-^13^C_3_,^15^N was used as a substrate (see Incorporation experiments in ESI†).

The hypothetical lincomycin intermediates **465** and **479** have their ‘detoxification label’ deacetylated, suggesting that deacetylation is involved in lincomycin maturation. Because we detected **479** as the major intermediate in the mutant strain of *S. lincolnensis* with an inactive *lmbF* gene, we considered that LmbF played a role in the subsequent conversion. Indeed, we detected significant consumption of **479** in the *in vitro* assay with recombinant LmbF (Fig. 5). The sequence analysis of LmbF shows that it belongs to the AAT_I (aspartate aminotransferase, fold type I) superfamily of enzymes, which employs pyridoxal-5′-phosphate (PLP) as a cofactor. Since a typical reaction for PLP-mediated turnovers is transamination (and with respect to celesticetin biosynthesis, as explained in the later text), we expected that LmbF would replace the primary amino group of **479** with the oxo functional group. Surprisingly, we did not detect a product corresponding to a simple transamination reaction. Instead, we found compound **392** (see Fig. 2D and 3) to be the product, which means that LmbF is responsible for the removal of the whole *S*-cysteine residue, *i.e.*, the complete deacetylated ‘detoxification label’ except for the sulfur atom (see LC-MS data in Fig. 5 and S6†). LmbF failed to carry out this reaction in the absence of PLP (Fig. S7†), confirming that the cofactor is essential for the turnover. Further, we observed that LmbF is also capable of removing the *S*-cysteine residue from **465** to give **378**, but less readily than from **479** (Fig. S6 and S7†), indicating that **479** is the main natural substrate of LmbF (this is also supported by the predominant presence of **479** over **465** in the culture broth of the strain with the inactive *lmbF* gene depicted in Fig. S8†). On the other hand, we did not detect any turnover of **507** or **521** (LC-MS data in Fig. 5 and S7A†) where the primary amino group is not available due to its acetylation.

**Fig. 6:**
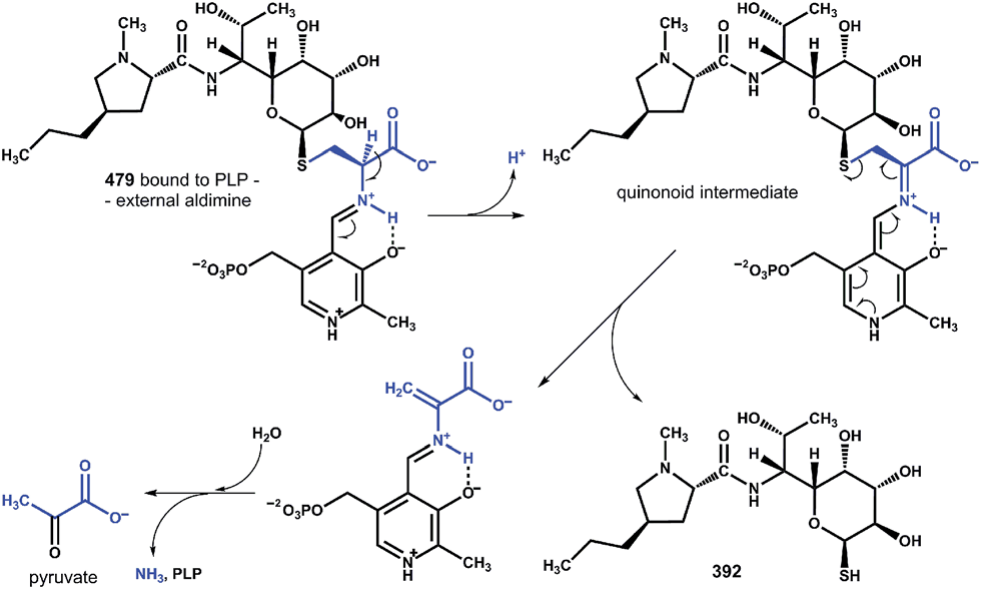
Proposed mechanism of β-elimination catalysed by PLP-dependent LmbF. The fate of the ‘detoxification label’ is shown in blue.

These findings provide insight into the reaction mechanism of the ‘detoxification label’ processing after it has been ‘unlocked’ by the deacetylation (Fig. 6): the unblocked primary amino group of **479** binds to PLP (a cofactor of LmbF) and LmbF catalyses the cleavage of the C–S bond by the β-elimination mechanism, which was described for PLP-dependent enzymes previously.^12^ This results in the release of **392** and pyruvate, which was also detected as a product of the reaction (Fig. 5).

### Reactive sulfhydryl group is protected by methylation

LmbF products **378** and **392** contain the exposed sulfhydryl group, which is prone to oxidation. Indeed, we observed a significant decrease of **378** and **392** along with the formation of dimers of these compounds (Fig. S9†) when the reaction time was prolonged from 2 h to 24 h. Therefore, we assume that the methylation of the sulfhydryl group occurs immediately after the removal of the *S*-cysteine residue to protect this reactive group. This reaction has been proposed to be catalysed by LmbG^3^ because it represents the only putative methyltransferase encoded within the lincomycin gene cluster whose function has not been assigned yet. The remaining two methyltransferases LmbW and LmbJ were proved to act as a *C*-methyltransferase^13^ and a *N*-methyltransferase,^6^ respectively, modifying the lincomycin amino acid unit.

### ‘Detoxification label’ abolishes antibacterial activity

Furthermore, we tested whether the intermediates **507**, **521**, **465** and **479** exhibit antibacterial activity against the lincomycin-sensitive Gram-positive bacterium *Kocuria rhizophila* and we compared the results with those for NDL and lincomycin. None of the intermediates containing the complete or deacetylated ‘detoxification label’ exhibited antibacterial properties against *K. rhizophila* (Fig. S10†). A comparison of the results for NDL and lincomycin shows that the *N*-methyl group enhances the antimicrobial activity; however, it is not crucial. On the other hand, the presence of the ‘detoxification label’ completely abolishes the antibiotic activity of the compound. This observation is in accordance with previous studies indicating that mercapturic acid derivatives are less bioactive compared to the parent antibiotic.^14^ Furthermore, not even deacetylation of the ‘detoxification label’ (intermediates **465** and **479**) is a sufficient condition for antimicrobial activity.

### Lincomycin/celesticetin biosynthesis: homologous LmbF/CcbF catalyse a different type of reaction, forming a branch point in the related pathways

**Fig. 7:**
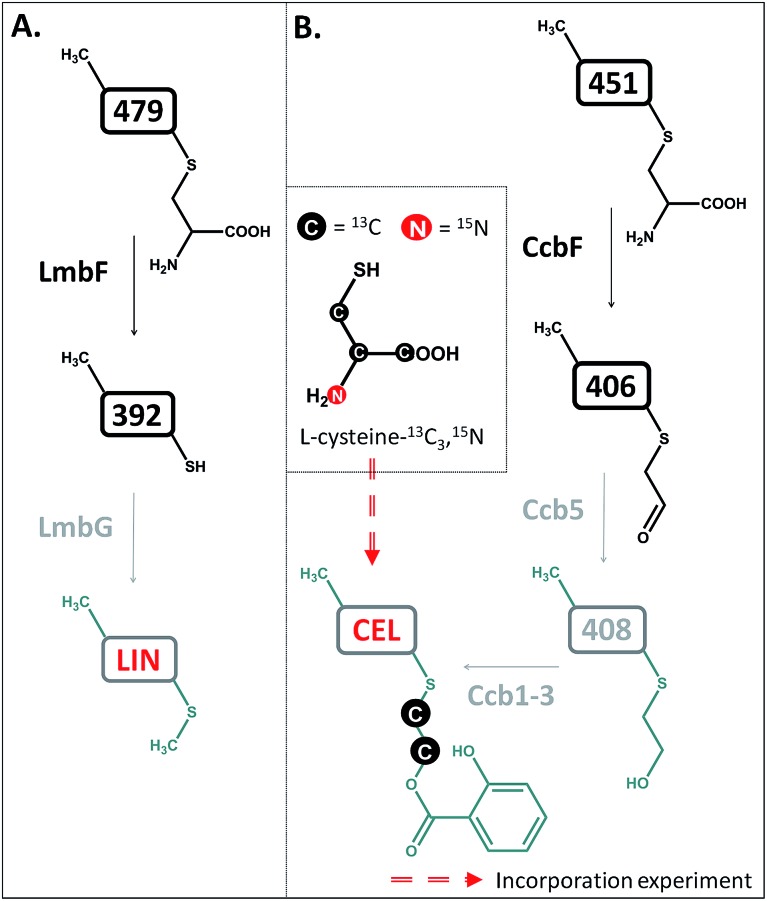
Different processing of the ‘detoxification label’ by homologous LmbF and CcbF (steps in black) in lincomycin (A) and celesticetin (B) biosyntheses. Incorporation of two ^13^C atoms from l-cysteine reveals the origin of the 2C linker of celesticetin – see Fig. S11† for MS data. LIN – lincomycin; CEL – celesticetin; the grey colour indicates deduced biosynthetic steps.

Lincomycin and celesticetin (Fig. 1) are the only known natural lincosamide antibiotics encoded by two independent but related biosynthetic gene clusters.^3,15^ In contrast to lincomycin, with its methylated sulfur atom, celesticetin bears salicylate attached to sulfur *via* a two-carbon (2C) linker. Particularly, the origin of the 2C linker was unclear until the lincomycin intermediate **507** was revealed. We assume that condensation of celesticetin proceeds through a mechanism analogous to that of lincomycin, *via* a hypothetical intermediate **451** (Fig. 7), which would be analogous to **479**; both compounds should bear the same deacetylated ‘detoxification label’. However, the fate of the ‘detoxification label’ is probably different in the biosynthesis of celesticetin and lincomycin (see the proposed scheme in Fig. 7). Based on the celesticetin structure, it can be assumed that the 2C linker remains from the *S*-cysteine residue. Indeed, we have confirmed that these two carbons originate from l-cysteine since we achieved 78% incorporation of two ^13^C atoms into the celesticetin 2C linker when the culture medium was supplemented with l-cysteine-^13^C_3_,^15^N (Fig. 7 and S11†). This means that CcbF does not catalyse the cleavage of the C–S bond by the β-elimination mechanism. Instead, CcbF presumably performs decarboxylation-dependent transamination, a reaction known to be catalysed by PLP-dependent enzymes as reviewed by John^16^ and documented on dialkylglycine decarboxylase, for example.^17^ The amino group would be replaced by an oxo group, which can be further reduced to a hydroxyl group (putative oxido-reductase encoded by the *ccb5* gene in the celesticetin gene cluster would be a suitable candidate for this reductase activity). Having completed this course of reactions, the intermediate is ready for conjugation with the acyl unit (already completed salicylate or its precursor) to form the final celesticetin scaffold (putative acyltransferase Ccb1, putative salicyl-AMP ligase Ccb2 and putative salicyl synthase Ccb3 can be expected to catalyse the formation and attachment of salicylate).

## Conclusion and perspectives

In this work, we present the final steps of lincomycin biosynthesis leading from the last proven intermediate, **507**, to lincomycin. Interestingly, we have demonstrated that LmbJ *N*-methylates a different substrate at an earlier stage of the biosynthesis than previously suggested. Furthermore, we have revealed that deacetylation of the ‘waste product’ **507** represents the key step that switches the detoxification-like MSH-dependent process towards the biosynthetic mode. Deacetylation provides a free primary amino group for the PLP-dependent LmbF responsible for processing the deacetylated ‘detoxification label’. PLP requires a primary amino group, to which it binds to enable any possible PLP-dependent turnovers including β-elimination in lincomycin biosynthesis. Furthermore, based on the structures of lincomycin and celesticetin and ^13^C incorporation experiments, we predict that homologous CcbF from celesticetin biosynthesis performs a different type of reaction, decarboxylation-dependent transamination, which results in preservation of the 2C residue allowing the attachment of the salicylate unit. Such a surprising finding that two homologous PLP-dependent proteins from highly related biosynthetic pathways should exhibit different reaction specificity undoubtedly represents an exciting topic of further studies.
