# Supplementary material for: Deacetylation of mycothiol-derived ‘waste product’ triggers the last biosynthetic steps of lincosamide antibiotics
Source: Chem Sci. 2015 Oct 1;7(1):430–5. doi: 10.1039/c5sc03327f (PMC5518657; doi:10.1039/c5sc03327f)
Supplement: Supplementary file 1 [file SC-007-C5SC03327F-s001.pdf]

Electronic Supplementary Material (ESI) for Chemical Science.
This journal is © The Royal Society of Chemistry 2015

# †Electronic supplemental information (ESI)

## Experimental

Bacterial strains

The *Streptomyces lincolnensis* mutant strains described below were derived from lincomycin producer *S. lincolnensis* ATCC 25466. *Streptomyces caelestis* ATCC 15084 was used as celesticetin producer. *Kocuria rhizophila* CCM 552 (Czech Collection of Microorganisms, Czech Republic; equivalent to ATCC 9341) was used as lincomycin sensitive strain for antibacterial activity assay. Routine DNA manipulations were performed in *E. coli* XL1 Blue MR[b] (Stratagene). The heterologous overproduction of *S. lincolnensis* proteins was performed in *E. coli* BL21(DE3) (Novagen). *E. coli* ET12567, BW25113 and DH5α strains were used for gene inactivation.

Gene inactivation

Generally, the inactivation of individual lincomycin biosynthetic genes was achieved by the Redirect targeting system[1] using the LK6 cosmid[2], which bears the whole lincomycin biosynthetic gene cluster. The resulting *S. lincolnensis* inactivation mutant strains were used mainly as a source of lincomycin biosynthesis intermediates (Δ*lmbIH* and optionally also Δ*lmbJ*). Only the production profile of *S. lincolnensis* Δ*lmbF* was used as evidence supporting the predicted order of reactions and the function of LmbF.

The inactivation mutant strains *S. lincolnensis* Δ*lmbJ* and Δ*lmbIH* (source of **507**, **521**, **465**, and **479**):

*S. lincolnensis* Δ*lmbIH* mutant strain with the inactivation apramycin cassette replaced by the 81 nucleotide long in-frame scar was constructed and described previously.[3] This strain served as a source of all four intermediates **507**, **521**, **465**, and **479** for NMR analysis and *in vitro* assays. The *lmbJ* gene was inactivated by introduction of an apramycin resistance cassette. Inactivation (dJf and dJr) and checking (cJf and cJr) primers for deletion of *lmbJ* are listed in Table S1. For dJf and dJr, the nucleotide extensions with sequence identity to LK6 are underlined, the nucleotides corresponding to start codons of *lmbJ* (in dJf) and *lmbIH* (in dJr) are marked in bold. In dJr, the nucleotides corresponding to *lmbJ* STOP codon are italicised. Due to the overlap of *lmbJ* and *lmbIH* genes the introduction of inactivation cassette to *lmbJ* logically disrupts also the expression of *lmbIH*. *S. lincolnensis* Δ*lmbJ* thus could serve as a source of **507** and **465** for *in vitro* assays (similarly to *S. lincolnensis* Δ*lmbIH*), but not of *N*-methylated **521** and **479** (because LmbJ is not produced).

The inactivation mutant strain *S. lincolnensis* Δ*lmbF* used as a supporting evidence of LmbF function

*S. lincolnensis* Δ*lmbF* mutant strain was constructed and described previously.[3] The introduction of inactivation apramycin cassette in *lmbF* could cause a polar effect on the downstream gene *lmbE*, however, as was documented by Zhao et al.,[4] the inactivation of *lmbE* is naturally partially compensated by action of another chromosomally encoded gene. We additionally compensated this possible partial polar effect by complementation carried out as follows. The *lmbE* gene was PCR amplified from the LK6 cosmid[2] using the primer pair LEf and LErstop (Table S1). The amplified sequence was inserted *via* the *Nde*I and *Xho*I restriction sites into an integrative vector pIJ10257.[5] The resulting construct was introduced into a non-methylating *E. coli* strain, ET12567/pUZ8002, and then introduced into the *S. lincolnensis* Δ*lmbF* genome *via* intergeneric conjugal transfer. Exconjugants were selected with hygromycin (0.1 $gL^{-1}$). The resulting *S. lincolnensis* mutant strain contained inactivation cassette instead of *lmbF* and beared extra copy of *lmbE* cloned into pIJ10257 under the control of the constitutive promoter *ermEp**. The vector was integrated in the genome at the ϕBT1 *attB* attachment site. Both mutant strains *S. lincolnensis* Δ*lmbF* (with and without extra copy of *lmbE* gene) accumulated identical compound **479**, differing only in the level of production (slightly higher in the *lmbE* compensated mutant presented here).

Table S1. Primers

| Name | Sequence (5′ to 3′) |
|---|---|
| LEf | CCGCATATGACTCAGTGCCTGCTGAC |
| LErstop | CCGCTCGAGTCACGCGGGAGCGGTGAACAG |
| LFf | CCGCATATGACCGCCACGGCGAGCGG |
| LFr | CCGCTCGAGCCGGTACCGCCACTCGGCC |
| dJf | CGAGTGGCCAACGTCCTGGTGAGGAAAGAGAATTCA**ATG**ATTCCGGGGATCCGTCGACC |
| dJr | GCGCCCTAG*T**CAC***CGTGCGCCCGGGCGTAGACGGACACGTGTAGGCTGGAGCTGCTTC |
| cJf | TCCCGGTCGAAGAACAC |
| cJr | GTCGCGCCCTAGTCAC |

Cultivation of streptomycete strains

The seed culture of *S. lincolnensis* or *S. caelestis* strains was prepared by inoculation of spores from MS plates into 50 mL of the YEME medium[6] without sucrose and incubated in 500 mL flat-bottom boiling flasks at 28 °C. Two mL of 24 h seed culture were inoculated into 40 mL of AVM[7] (*S. lincolnensis*) or GYM[8] medium (*S. caelestis*) and incubated in 500 mL flat-bottom boiling flasks at 28 °C for 120 h. The cells were centrifuged at 5000 g at 20 °C for 15 min and the supernatant was used for LC-MS analyses or purification of lincomycin derivatives.

L-cysteine-$^{13}C_3$,$^{15}N$ incorporation experiments

*Cultivation.* GYM cultivation broth after inoculation by the seed culture of *S. caelestis* was supplemented with 200mM L-cysteine-$^{13}C_3$,$^{15}N$ (95%, labelling 98%, Sigma-Aldrich, Germany) as follows: 250 µL after 36 h from inoculation, 500 µL (48 h), 750 µL (60, 72, and 84 h). AVM cultivation broth after inoculation by seed culture of the Δ*lmbIH* mutant strain of *S. lincolnensis* was supplemented with the same chemical as follows: 80 µL (36, and 72 h). After 120 h the cells were centrifuged at 5000 g at 20 °C for 15 min and the supernatant was used for extraction.

*Extraction*. An Oasis HLB 3cc 60 mg cartridge (hydrophilic lipophilic balanced sorbent, Waters, USA) was conditioned with 3 mL methanol (LC-MS grade, Biosolve, Netherlands), equilibrated with 3 mL water, and then 3 mL cultivation broth (pH adjusted to 9 with ammonium hydroxide, 28-30%, Sigma-Aldrich, Germany) was loaded. Subsequently, the cartridge was washed with 3 mL water, and absorbed substances were eluted with 1.5 mL methanol. The eluent was evaporated to dryness, reconstituted in 1.5 mL methanol and analysed by LC-MS as described in the paragraph LC-MS analyses.

*Detection of $^{13}C_3$-pyruvate, the product of in vitro reaction with **479**-$^{13}C_3$,$^{15}N$.* The extract (100 µL) of the Δ*lmbIH* mutant strain of *S. lincolnensis* supplemented with L-cysteine-$^{13}C_3$,$^{15}N$ was repeatedly injected into LC-MS (see the paragraph LC-MS analyses) and the column effluent containing the mixture of **479** and **479**-$^{13}C_3$,$^{15}N$ was collected before entering the MS detector. The column effluent was evaporated to dryness, reconstituted in 30 µL water and 10 µL of it were used for the *in vitro* assay with LmbF as described in the paragraph *In vitro* assays. The 50 µL reaction or authentic standard of pyruvic acid (Sigma-Aldrich, Germany; used for retention time comparison – data not shown) was mixed with 50 µL of 2,4-dinitrophenylhydrazine solution (1 mg $mL^{-1}$ in 2M HCl, TCI America, USA), incubated at 30 °C for 2 h, neutralised with ammonium hydroxide and analysed by LC-MS as described in the paragraph LC-MS analyses except for the following conditions: the two-component

mobile phase consisted of a mobile phase, A and B, consisting of 0.1% formic acid (98-100%, Merck, Germany) and acetonitrile (LC-MS grade, Biosolve, Netherlands), respectively. The analyses were performed at 40 °C under a linear gradient program (min/%B) 0/5, 1.5/5, 15/70, 18/99 followed by a 1-min column clean-up (99% B) and 1.5-min equilibration (5% B), at the flow rate of 0.4 mL $min^{-1}$. The mass spectra were measured in the negative mode with capillary voltage set at −2600 V, cone voltage −40 V.

Assay of antibacterial properties

Filtration paper discs (diameter, 5 mm) containing the tested compounds were transferred onto plates overlaid with the lincomycin-sensitive strain *K. rhizophila*. The plates were cultivated at 37 °C overnight, and the antibacterial properties were detected by growth inhibition zones around discs.

Purification and quantification of lincomycin derivatives for *in vitro* assays

*Extraction:* Compounds **465** and **507** were purified from the culture broth of the Δ*lmbJ* mutant strain, and compounds **479** and **521** were purified from the culture broth of the Δ*lmbIH* mutant strain. An Oasis MCX 6cc 200 mg cartridge (mixed-mode cation exchange sorbent, Waters, USA) was conditioned with 5 mL methanol, equilibrated with 5 mL 2 % formic acid, and then 50 ml cultivation broth (pH adjusted to 2.3 with formic acid) was loaded. Subsequently, the cartridge was washed with 5 mL 2% formic acid and 5 mL methanol, and absorbed substances were eluted with 15 mL of a methanol:water:ammonium hydroxide solution (50:48.5:1.5 v/v/v). The eluent was evaporated to dryness, reconstituted in 2 mL methanol and used for HPLC purification.

*HPLC purification:* The extracts were injected into the HPLC apparatus equipped with flow controller 600, autosampler 717, and UV detector 2487 operating at 194 nm (Waters, USA). Data were processed with Empower 2 software (Waters, USA). The analytes were separated on the Luna C18 chromatographic column (250 × 15 mm I.D., particle size 5 µm, Phenomenex, USA) with the two component mobile phase, A and B, consisting of 0.1% formic acid and methanol, respectively. The analyses were performed under a linear gradient program (min/%B) 0/5, 31/27.5 followed by a 9-min column clean-up (100% B) and a 9-min equilibration (5% B), at a flow rate of 3 mL $min^{-1}$. The fractions containing lincomycin derivatives were evaporated to dryness, reconstituted in methanol and injected onto the XTerra Prep RP18 column (150 × 7.8 mm I.D., particle size 5.0 µm, Waters, USA). The lincomycin derivatives were eluted using the isocratic program with 1 mM ammonium formate (pH 9.0):acetonitrile (90:10 v/v) as mobile phase. The fractions containing the separated lincomycin derivatives were checked for purity (>95% - LC with UV detection at 220 nm; no significant cross-contamination of the lincomycin derivatives).

*Quantification:* The HPLC-purified lincomycin derivatives were reconstituted in water and analysed using the same chromatographic conditions as described below for LC-MS analyses. The column effluent was monitored by UV detection and the response at 194 nm was used for quantification. Compounds **465** and **507** were quantified using the calibration curve of *N*-demethyllincomycin (synthesized as described in the literature[9]), and compounds **479** and **521** were quantified using the calibration curve of lincomycin (hydrochloride, Sigma-Aldrich, Germany). The six calibration points in the 12.5-500 µg $ml^{-1}$ range were fitted linearly with the determination coefficient of 0.9987 for *N*-demethyllincomycin and 0.9996 for lincomycin. The method used was a modification of a validated method for determination of lincomycin.[10]

Large-scale purification of lincomycin derivatives and nuclear magnetic resonance (NMR) experiments

The intermediates **507**, **521**, **465**, and **479** were purified from 800 mL of culture broth of the Δ*lmbIH* mutant strain. The culture broth adjusted to pH 9 with ammonium hydroxide was loaded onto 10 g of Amberlite XAD-4 (Supelco, USA) pre-conditioned with 100 mL methanol and 100 mL water. The sorbent was then washed with 200 mL ammonium formate diluted to pH 9. Absorbed intermediates were subsequently eluted with methanol-water solvent of increasing methanol ratio: from methanol-water 10:90 (v/v) up to 80:20 (v/v) with the steps of 10 % and volume of each solvent of 100 mL. Fractions containing the intermediates of interest (guided by LC-

MS as described below in the paragraph LC-MS analyses) were pre-concentrated using speedvac and subjected to HPLC purification as it is described above in the paragraph dealing with purification for *in vitro* assays). Purified intermediates (**507**: 2.0 mg, **521**: 1.2 mg, **465**: 0.9 mg, **479**: 0.8 mg) were analysed by NMR. NMR spectra were recorded on a Bruker Avance III 700 MHz spectrometer (700.13 MHz for $^{1}$H, 176.05 MHz for $^{13}$C) in $CD_3OD$ (100.0 atom% D, Sigma Aldrich, Steinheim, Germany) at 20 °C. Residual signals of solvent were used as an internal standard ($\delta_H$ 3.330, $\delta_C$ 49.30). NMR experiments: $^{1}$H NMR, $^{13}$C NMR, COSY, HSQC, HMBC, and 1D TOCSY were performed using the manufacturer's software. $^{1}$H NMR and $^{13}$C NMR spectra were zero filled to fourfold data points and multiplied by window function before Fourier transformation. Two-parameter double-exponential Lorentz-Gauss function was applied for $^{1}$H to improve resolution and line broadening (1 Hz) was applied to get better $^{13}$C signal-to-noise ratio. Chemical shifts are given in $\delta$-scale with digital resolution justifying the reported values to three ($\delta_H$) or two ($\delta_C$). Coupling constants are in Hz. Some carbon chemical shifts were read out from HSQC and HMBC spectra and they are given to one decimal place.

Heterologous production and purification of LmbF and LmbJ

Gene *lmbF* was PCR amplified from LK6 cosmid[2] using the primer pair LFf and LFr (Table S1) and inserted into pET42 vector (Novagen). The resulting construct was used to produce soluble C-terminally His-tagged fusion protein LmbF in *E. coli* BL21(DE3) with co-expression with GroES and GroEL chaperonins. The protein was produced and purified as was described for LmbC[11] with additional 20% (v/v) glycerol in all buffers. The buffer in the protein sample was then exchanged on a HiTrap™ Desalting Column (GE Healthcare) to 20 mM Tris, 100 mM NaCl, 20% (v/v) glycerol (pH 8.0) and the protein was stored at –20 °C.

N-terminally His-tagged fusion protein LmbJ was overproduced as described in the literature.[12]

Bioinformatic tools

The BLASTX and BLASTP were used for prediction of putative functions of encoded proteins (http://blast.ncbi.nlm.nih.gov/Blast.cgi)[13] in combination with Conserved Domains Database (http://www.ncbi.nlm.nih.gov/cdd).[14]

*In vitro* assays

*Assay with LmbJ.* The substrates **465**, **507** and NDL were tested separately in the following reactions: 50 µM substrate, 2 mM *S*-adenosyl-methionine (SAM, 80%, Sigma-Aldrich, Germany), 10 µg LmbJ; reaction volume, 40 µL in 100 mM Tris buffer pH 8.0; temperature, 30 °C; time, 120 min.

*Competitive assay with LmbJ.* The substrates **465**, **507** and NDL were tested simultaneously in one reaction mixture: 50 µM substrates (each), 2 mM SAM, 30 µg LmbJ; reaction volume, 120 µL in 100 mM Tris buffer pH 8.0; temperature, 30 °C; aliquots of reactions were terminated after 5, 10, 30, 60, 120, and 240 min. A negative control was used for the time point of 0 min.

*Assay with LmbF.* The substrates **465**, **507**, **479**, and **521** were tested separately in the following reactions: 50 µM substrate, 50 µM pyridoxal-5′-phosphate (PLP, >98%, Sigma-Aldrich, Germany), 2 µg LmbF, 100 mM Tris buffer pH 8.0; reaction volume, 50 µL; temperature, 30 °C; time, 120 min. PLP dependence of LmbF was tested by replacing PLP solution with water.

Negative controls were performed with proteins inactivated by heat (80 °C, 10 min). Reactions were terminated by adding an equal volume of acetonitrile, centrifuged (3000 g, 5 min) and analysed by LC-MS. Samples of the competitive assay with LmbJ were prior analysis supplemented with 20 µM 4′-butyl-4′-depropyllincomycin (prepared as described in the literature[15]) used as internal standard to compensate for MS signal fluctuations.

LC-MS analyses

LC-MS analyses were performed on the Acquity UPLC system with LCT premier XE time-of-flight mass spectrometer (Waters, USA). Five µL of sample was loaded onto the Acquity UPLC BEH C18 LC column (50 mm × 2.1 mm I.D., particle size 1.7 µm, Waters) kept at 30 °C and eluted with a two-component mobile phase, A and B, consisting of 1 mM ammonium formate, pH 9.0 and acetonitrile, respectively. The analyses were performed under a linear gradient program (min/%B) 0/5, 1.5/5, 12.5/58 followed by a 2-min column clean-up (100% B) and 1.5-min equilibration (5% B), at the flow rate of 0.4 mL min$^{-1}$. The mass spectrometer operated in the "W" mode with capillary voltage set at +2800 V, cone voltage +40 V, desolvation gas temperature, 350 °C; ion source block temperature, 120 °C; cone gas flow, 50 Lh$^{-1}$; desolvation gas flow, 800 Lh$^{-1}$; scan time of 0.1 s; inter-scan delay of 0.01 s. The mass accuracy was kept below 5 ppm using lock spray technology with leucine enkephalin as the reference compound (2 ng µl$^{-1}$, 5 µl min$^{-1}$). Fragmentation by collision-induced dissociation (CID) was triggered by increasing aperture 1 at 50 V. Chromatograms were extracted for $[M+H]^+$ ions with the tolerance window of 0.05 Da. The data were processed by MassLynx V4.1 (Waters).

**507**

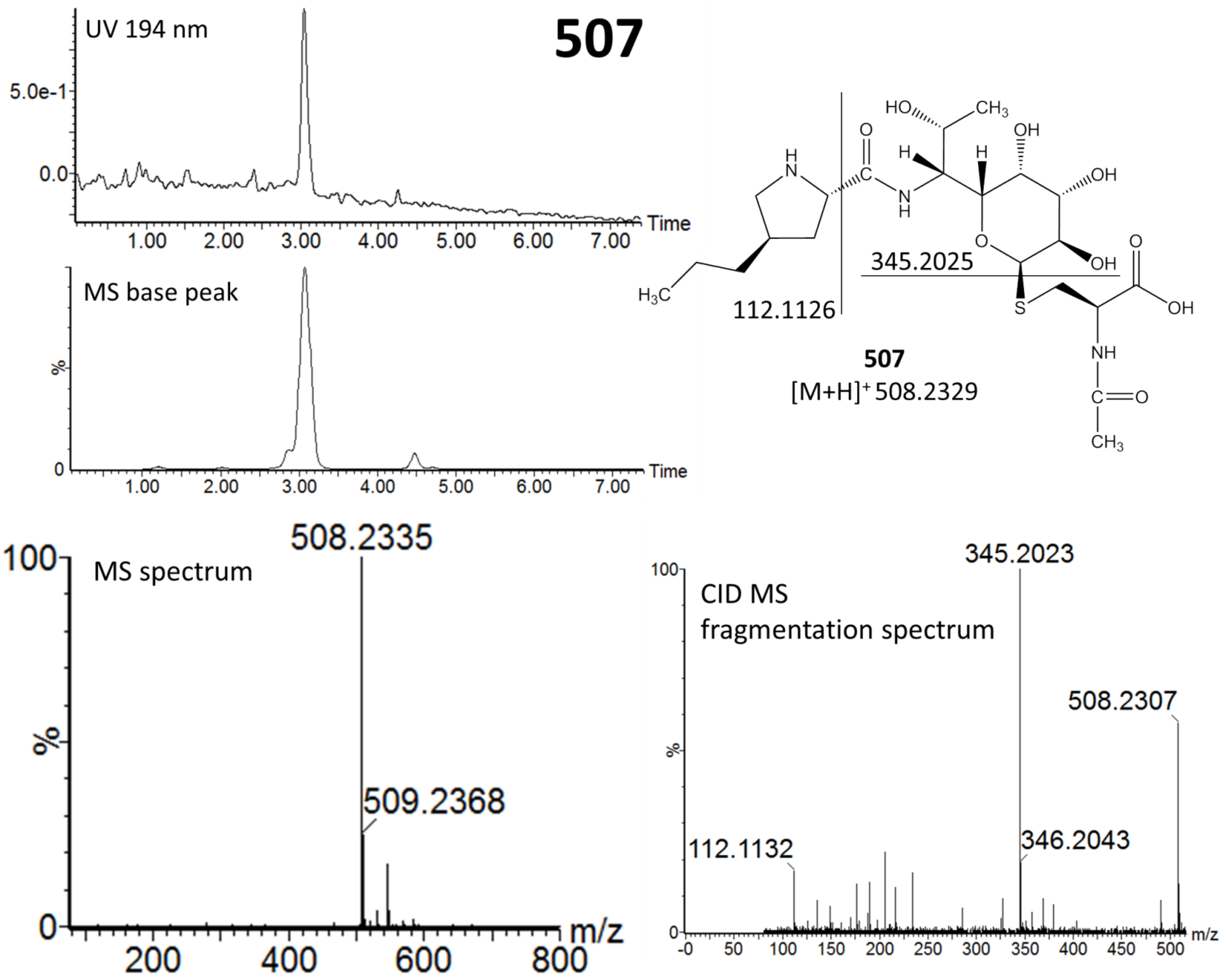
UV 194 nm
5.0e-1
0.0
1.00 2.00 3.00 4.00 5.00 6.00 7.00 Time
MS base peak
%
0
1.00 2.00 3.00 4.00 5.00 6.00 7.00 Time
345.2025
112.1126
507
[M+H]+ 508.2329
MS spectrum
508.2335
509.2368
100
%
0
200 400 600 800 m/z
CID MS
fragmentation spectrum
345.2023
508.2307
346.2043
112.1132
100
%
0
-0 50 100 150 200 250 300 350 400 450 500 m/z

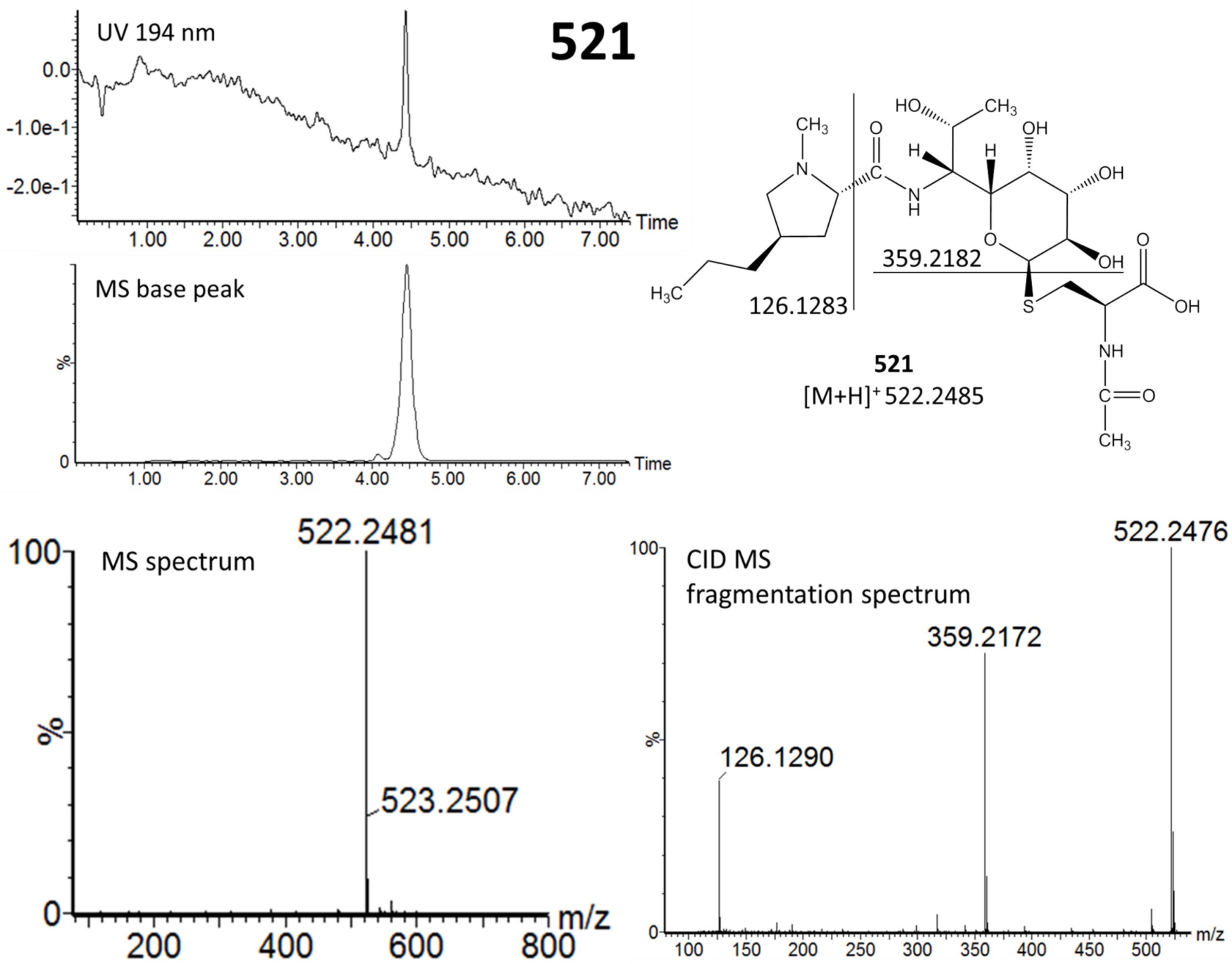
521
UV 194 nm
0.0
-1.0e-1
-2.0e-1
Time
MS base peak
%
126.1283
359.2182
521
[M+H]+ 522.2485
MS spectrum
522.2481
523.2507
m/z
CID MS
fragmentation spectrum
522.2476
359.2172
126.1290

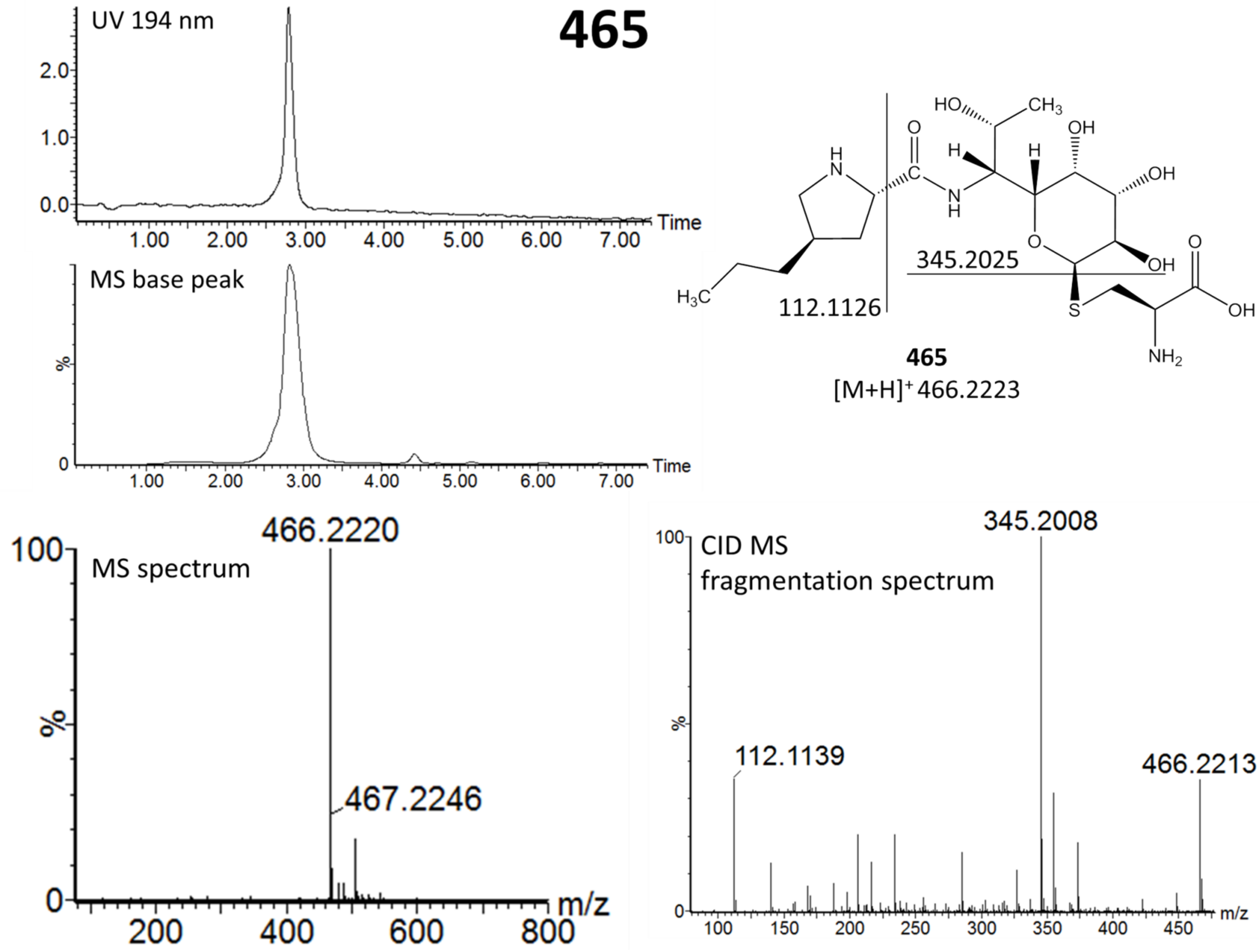
465
UV 194 nm
2.0
1.0
0.0
1.00 2.00 3.00 4.00 5.00 6.00 7.00
Time
MS base peak
%
0
1.00 2.00 3.00 4.00 5.00 6.00 7.00
Time
HO
CH3
O
OH
H
H
H
N
C
OH
N
H
O
O
345.2025
OH
H3C
112.1126
S
OH
NH2
465
[M+H]+ 466.2223
100
MS spectrum
466.2220
%
467.2246
0
200 400 600 800
m/z
100
CID MS
fragmentation spectrum
345.2008
%
112.1139
466.2213
0
100 150 200 250 300 350 400 450
m/z

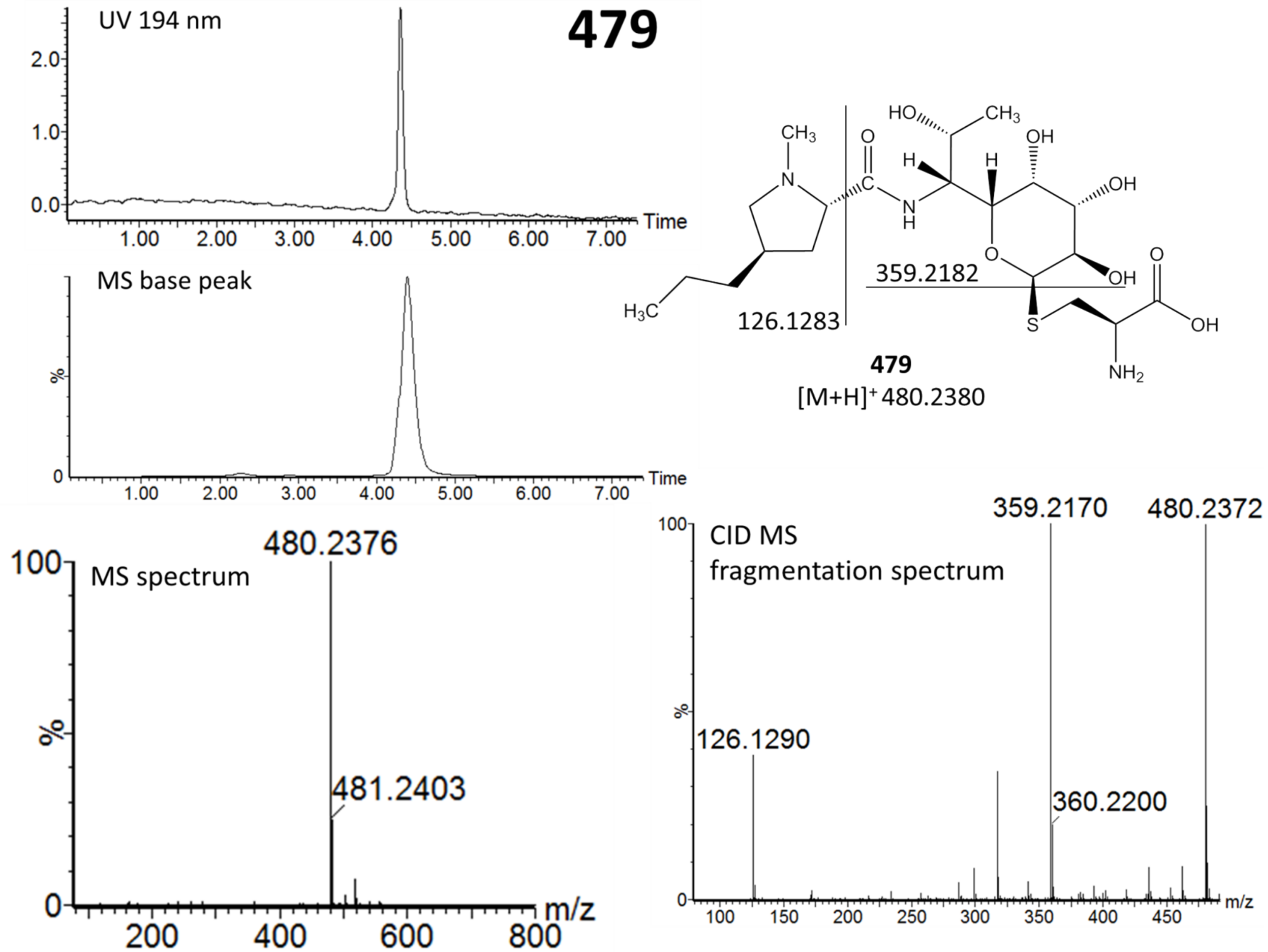

479
UV 194 nm
2.0
1.0
0.0
Time
1.00
2.00
3.00
4.00
5.00
6.00
7.00
MS base peak
%
0
126.1283
359.2182
479
[M+H]+ 480.2380
MS spectrum
480.2376
481.2403
100
0
200
400
600
800
m/z
CID MS
fragmentation spectrum
359.2170
480.2372
126.1290
360.2200
100
150
250
300
350
450

# NDL

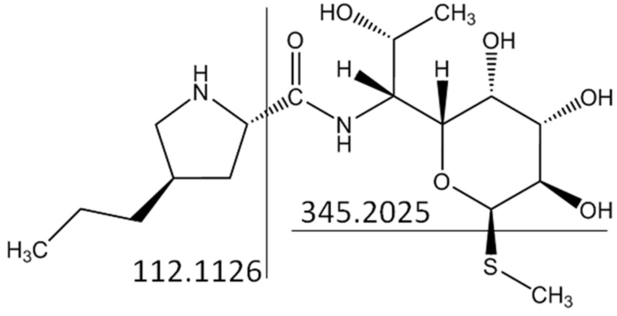


**NDL**
$[M+H]^+$ 393.2059

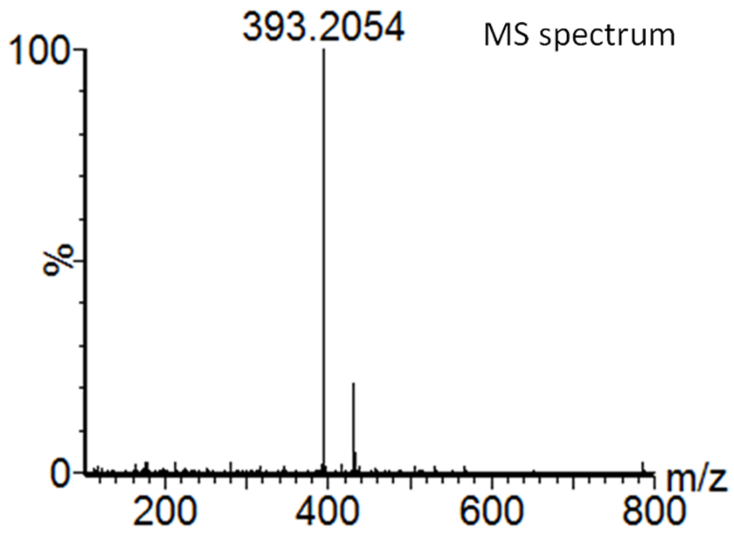


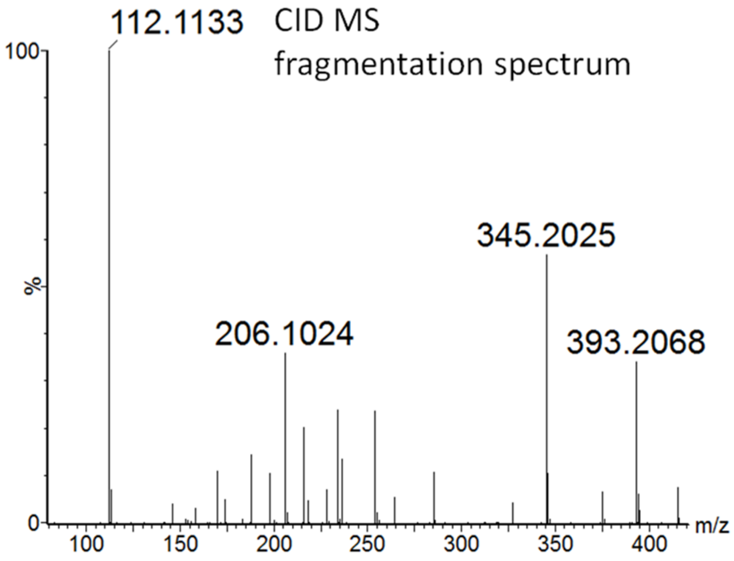

# LIN

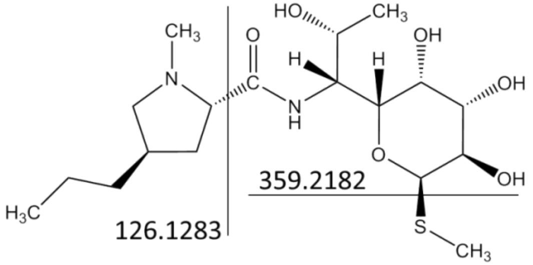


**LIN**
$[M+H]^+$ 407.2216

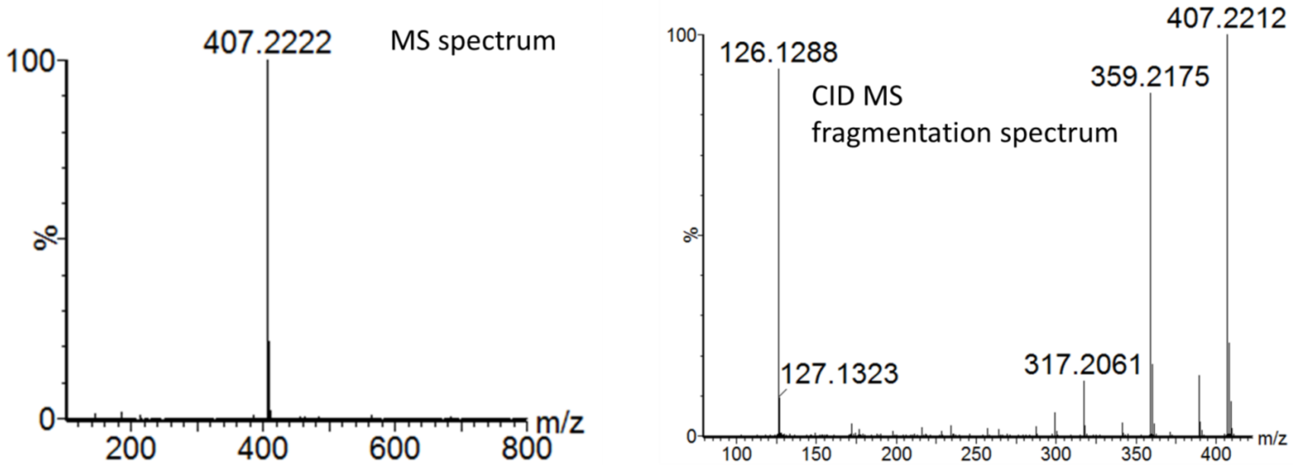


**Fig. S1** Lincomycin intermediates purified from culture broths (compounds **507**, **521**, **465**, and **479**). UV (194 nm) and MS base peak chromatograms after purification; MS and in-source CID MS fragmentation spectra of the compounds. MS and in-source CID MS characteristics of *N*-demethyllincomycin (NDL) and lincomycin (LIN) authentic standards.

**Table S2** NMR data of lincomycin intermediates **465**, **479**, **507**, and **521:** A. $^{1}$H NMR data (700.13 MHz, $CD_3OD$, 20 °C); B. $^{13}$C NMR data (176.05 MHz, $CD_3OD$, 20 °C).

A.

| | **465** | | | **479** | | | **507** | | | **521** | | |
|---|---|---|---|---|---|---|---|---|---|---|---|---|
| position | $\delta_H$ | m. | $J_{HH}$ [Hz] | $\delta_H$ | m. | $J_{HH}$ [Hz] | $\delta_H$ | m. | $J_{HH}$ [Hz] | $\delta_H$ | m. | $J_{HH}$ [Hz] |
| 1 | 5.408 | d | 5.6 | 5.373 | d | 5.5 | 5.466 | d | 5.7 | 5.456 | d | 5.6 |
| 2 | 4.119 | dd | 5.6, 10.2 | 4.127 | dd | 5.5, 10.3 | 4.104 | dd | 5.7, 10.1 | 4.096 | dd | 5.6, 10.2 |
| 3 | 3.528 | dd | 3.3, 10.2 | 3.528 | dd | 3.3, 10.3 | 3.546 | dd | 3.2, 10.1 | 3.555 | dd | 3.3, 10.2 |
| 4 | 4.018 | dd | 1.3, 3.3 | 4.077 | dd | 1.4, 3.3 | 3.936 | dd | 1.1, 3.2 | 3.941 | dd | 1.0, 3.3 |
| 5 | 4.317 | dd | 1.3, 6.9 | 4.398 | dd | 1.4, 6.3 | 4.246 | dd | 1.1, 5.1 | 4.257 | dd | 1.0, 6.4 |
| 6 | 4.236 | dd | 6.9, 7.2 | 4.231 | dd | 6.3, 8.0 | 4.199 | dd | 5.1, 7.0 | 4.276 | dd | 6.3,6.4 |
| 7 | 3.984 | dq | 6.4, 7.2 | 3.930 | dq | 6.3, 8.0 | 4.144 | dq | 6.3, 7.0 | 4.083 | dq | 6.3, 6.4 |
| 8 | 1.230 | d | 6.4 | 1.247 | d | 6.3 | 1.210 | d | 6.3 | 1.209 | d | 6.4 |
| N-$CH_3$ | - | | | 2.374 | s | - | - | | | 2.727 | s | - |
| 2’ | 3.85* | m | - | 2.940 | dd | 4.5, 10.6 | 4.435 | dd | 5.0, 9.0 | 3.761 | br s | - |
| 3’a | 2.072 | ddd | 4.0, 7.1, 12.5 | 2.004 | ddd | 4.5, 8.1, 12.7 | 2.237 | ddd | 5.0, 7.4, 13.2 | 2.172 | ddd | 5.6, 8.1, 13.4 |
| 3’b | 1.788 | ddd | 9.2, 9.2, 12.5 | 1.809 | ddd | | 2.068 | ddd | 9.0, 9.0, 13.2 | 2.087 | m | - |
| 4’ | 2.139 | m | - | 2.259 | m | - | 2.358 | m | - | 2.321 | m | - |
| 5’a | 3.24* | m | - | 3.200 | dd | 6.0, 8.5 | 3.558 | dd | 7.3, 11.1 | 3.540 | m | - |
| 5’b | 2.609 | dd | 8.6, 9.9 | 2.071 | dd | 8.5, 10.3 | 2.911 | dd | 9.3, 11.1 | 2.569 | m | - |
| 6’ | 1.375 | m | - | 1.346 | m | - | 1.461 | m | - | 1.450 | m | - |
| 7’ | 1.375 | m | - | 1.346 | m | - | 1.396 | m | - | 1.368 | m | - |
| 8’ | 0.954 | m | - | 0.944 | m | - | 0.973 | t | 7.2 | 0.960 | t | 7.2 |
| 1’’a | 3.184 | dd | 6.9, 14.8 | 3.221 | dd | 6.9, 14.9 | 3.063 | dd | 4.5, 13.1 | 3.081 | dd | 4.6, 13.4 |
| 1’’b | 3.129 | dd | 3.8, 14.8 | 3.127 | dd | 3.5, 14.9 | 3.001 | dd | 7.5, 13.1 | 3.043 | dd | 6.6, 13.4 |
| 2’’ | 3.785 | dd | 3.8, 6.9 | 3.820 | dd | 3.5, 6.9 | 4.370 | dd | 4.5, 7.5 | 4.482 | dd | 4.6, 6.6 |
| Ac | - | | | - | | | 2.014 | s | - | 2.018 | s | - |

a, b – diastereotopic methylene protons, a – proton at lower field, b – proton at the higher field.

B.

| | 465 | | 479 | | 507 | | 521 | |
|---|---|---|---|---|---|---|---|---|
| position | $\delta_C$ | m. | $\delta_C$ | m. | $\delta_C$ | m. | $\delta_C$ | m. |
| 1 | 90.30 | d | 90.61 | d | 89.44 | d | 89.42 | d |
| 2 | 69.61 | d | 69.62 | d | 69.61 | d | 69.68 | d |
| 3 | 72.09 | d | 72.00 | d | 72.37 | d | 72.22 | d |
| 4 | 70.90 | d | 70.96 | d | 71.51 | d | 71.24 | d |
| 5 | 71.79 | d | 71.61 | d | 73.04 | d | 72.11 | d |
| 6 | 56.70 | d | 56.69 | d | 57.97 | d | 57.05 | d |
| 7 | 68.35 | d | 68.64 | d | 66.86 | d | 67.68 | d |
| 8 | 20.21 | q | 20.81 | q | 19.85 | q | 19.57 | q |
| N-$CH_3$ | - | | 42.07 | q | - | | 41.62 | q |
| 1’ | 177.5[H] | s | 178.47 | s | 170.56 | s | 172.9[H] | s |
| 2’ | 61.67 | d | 70.40 | d | 61.14 | d | 70.08 | d |
| 3’ | 38.69 | t | 39.14 | t | 37.18 | t | 37.67 | t |
| 4’ | 39.87 | d | 39.26 | d | 39.00 | d | 38.66 | d |
| 5’ | 53.73 | t | 64.13 | t | 52.43 | t | 63.17 | t |
| 6’ | 36.78 | t | 37.24 | t | 35.76 | t | 36.62 | t |
| 7’ | 23.07 | t | 23.03 | t | 22.58 | t | 22.64 | t |
| 8’ | 14.90 | q | 14.94 | q | 14.60 | q | 14.71 | q |
| 1’’ | 34.24 | t | 34.51 | t | 34.60 | t | 34.56 | t |
| 2’’ | 56.46 | d | 56.48 | d | 57.04 | d | 56.43 | d |
| 3’’ | 173.1[H] | s | 172.67 | s | 177.33 | s | 176.72 | s |
| C=O | - | | - | | 173.09 | s | 173.00 | s |
| Ac | - | | - | | 23.11 | q | 23.14 | q |

[H] – HMBC readout

**A.**

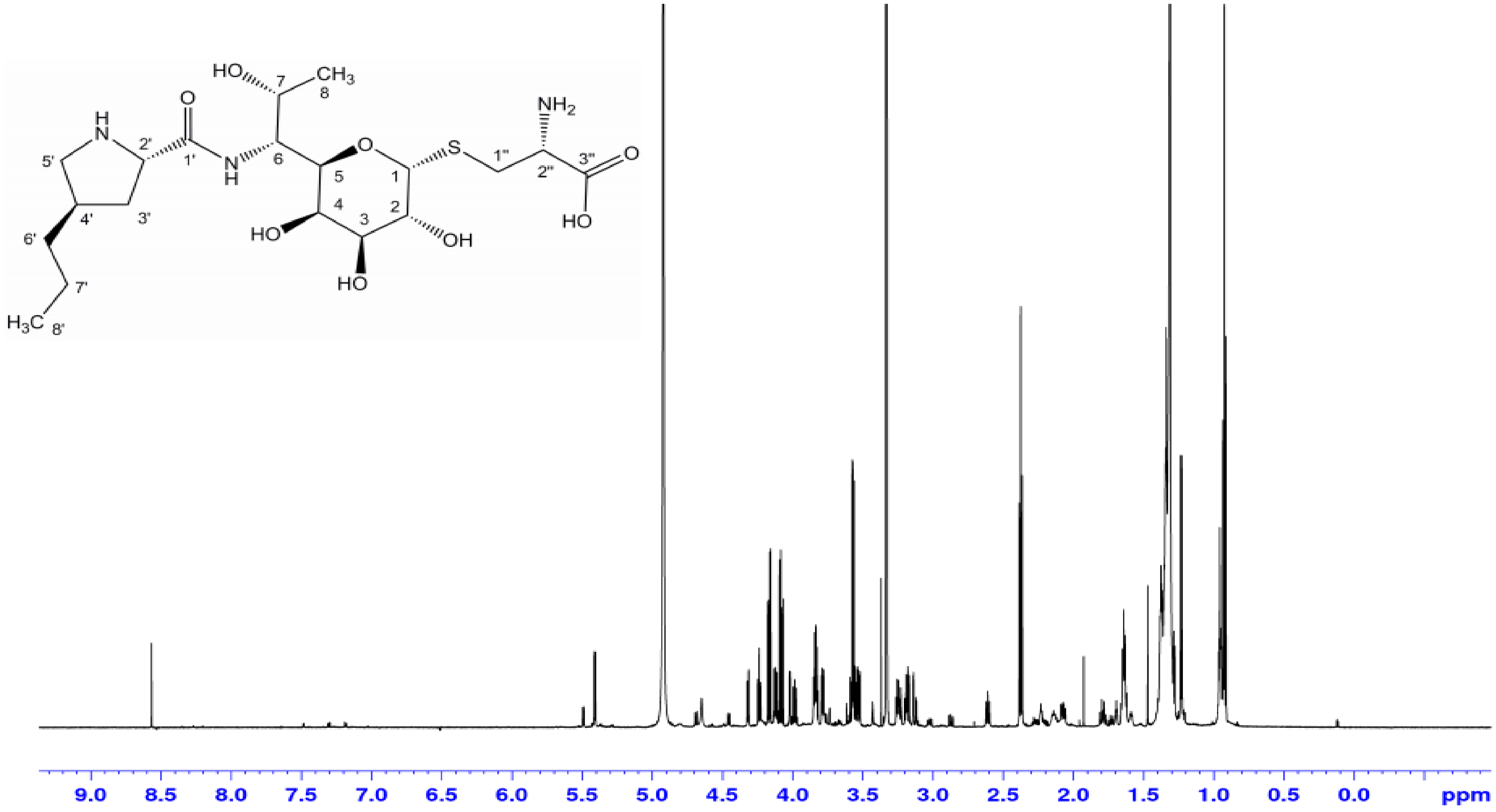

HO
7
$CH_3$
8
$NH_2$
O
H
N
2'
1'
5'
N
H
6
O
5
1
S
1''
2''
3''
O
HO
4'
3'
4
3
2
HO
OH
HO
6'
7'
$H_3C$
8'
9.0
8.5
8.0
7.5
7.0
6.5
6.0
5.5
5.0
4.5
4.0
3.5
3.0
2.5
2.0
1.5
1.0
0.5
0.0
ppm

**B.**

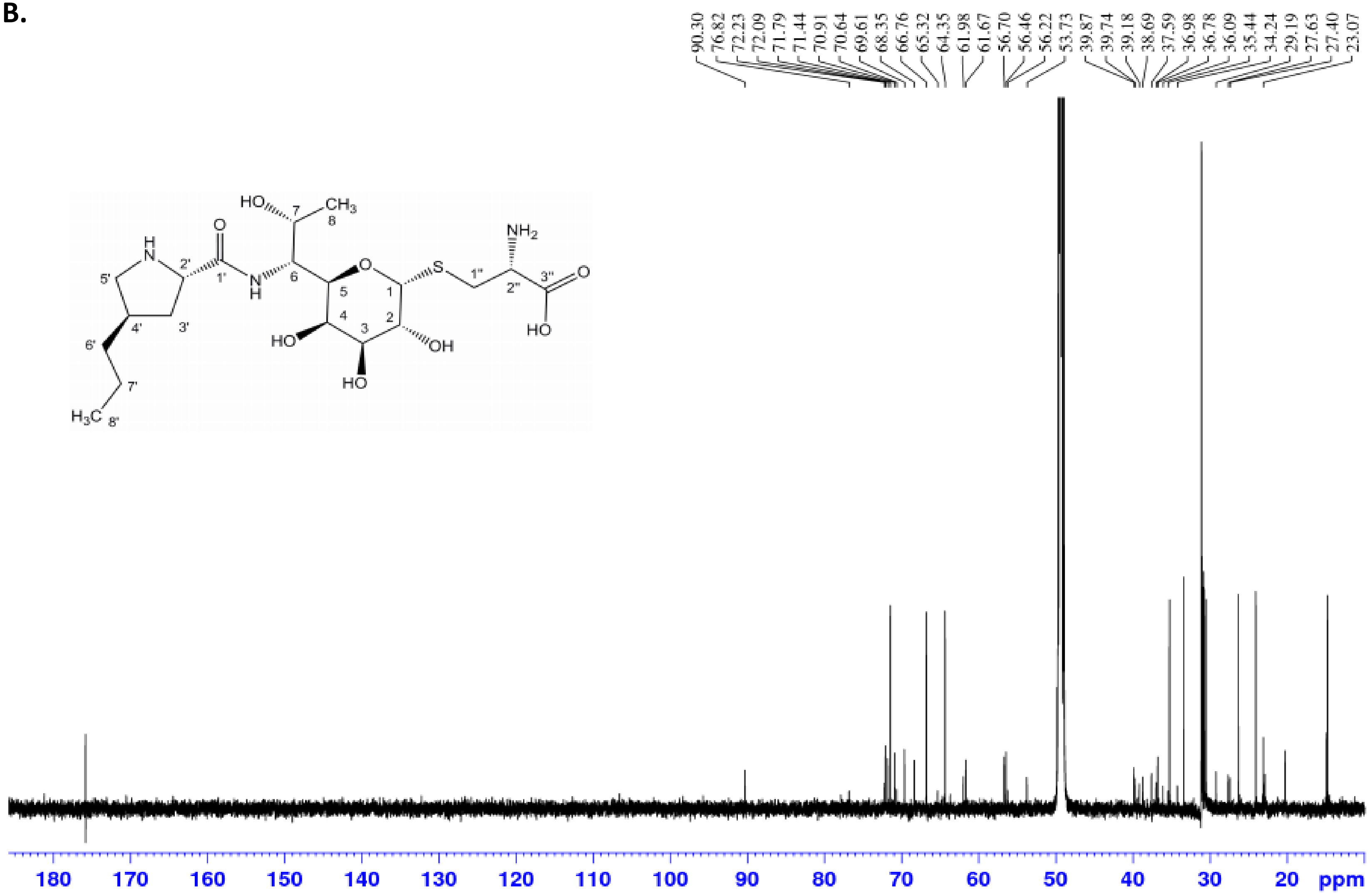

90.30
76.82
72.23
72.09
71.79
71.44
70.91
70.64
69.61
68.35
66.76
65.32
64.35
61.98
61.67
56.70
56.46
56.22
53.73
39.87
39.74
39.18
38.69
37.59
36.98
36.78
36.09
35.44
34.24
29.19
27.63
27.40
23.07
HO
CH3
NH2
O
S
HO
OH
H3C
180
170
160
150
140
130
120
110
100
90
80
70
60
50
40
30
20
ppm

C.

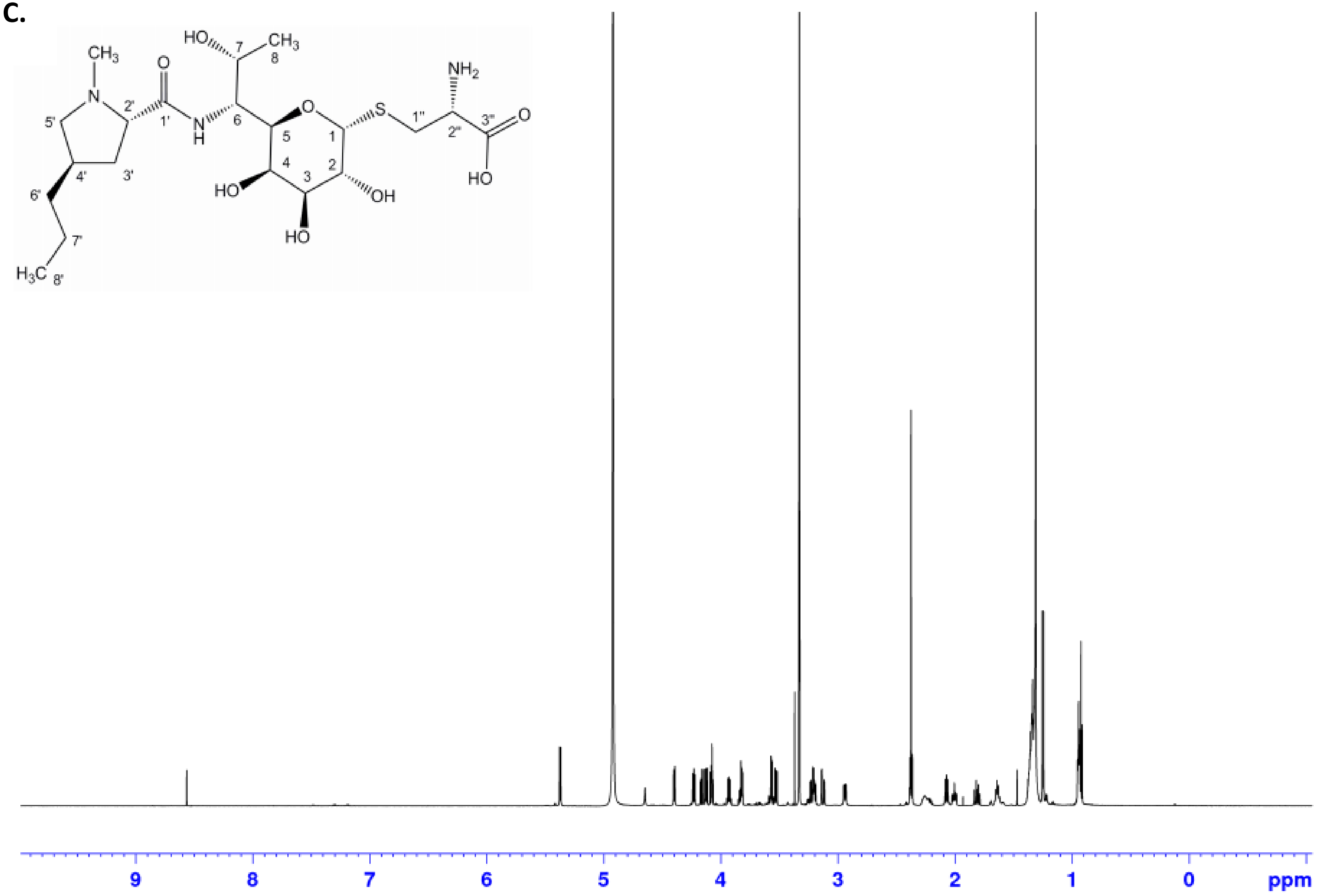

CH3
HO
7
CH3
8
NH2
O
N
2'
5'
1'
N
H
6
5
O
1
S
1''
2''
3''
O
HO
4
3
2
4'
3'
6'
HO
OH
HO
7'
H3C
8'
9
8
7
6
5
4
3
2
1
0
ppm

**D.**

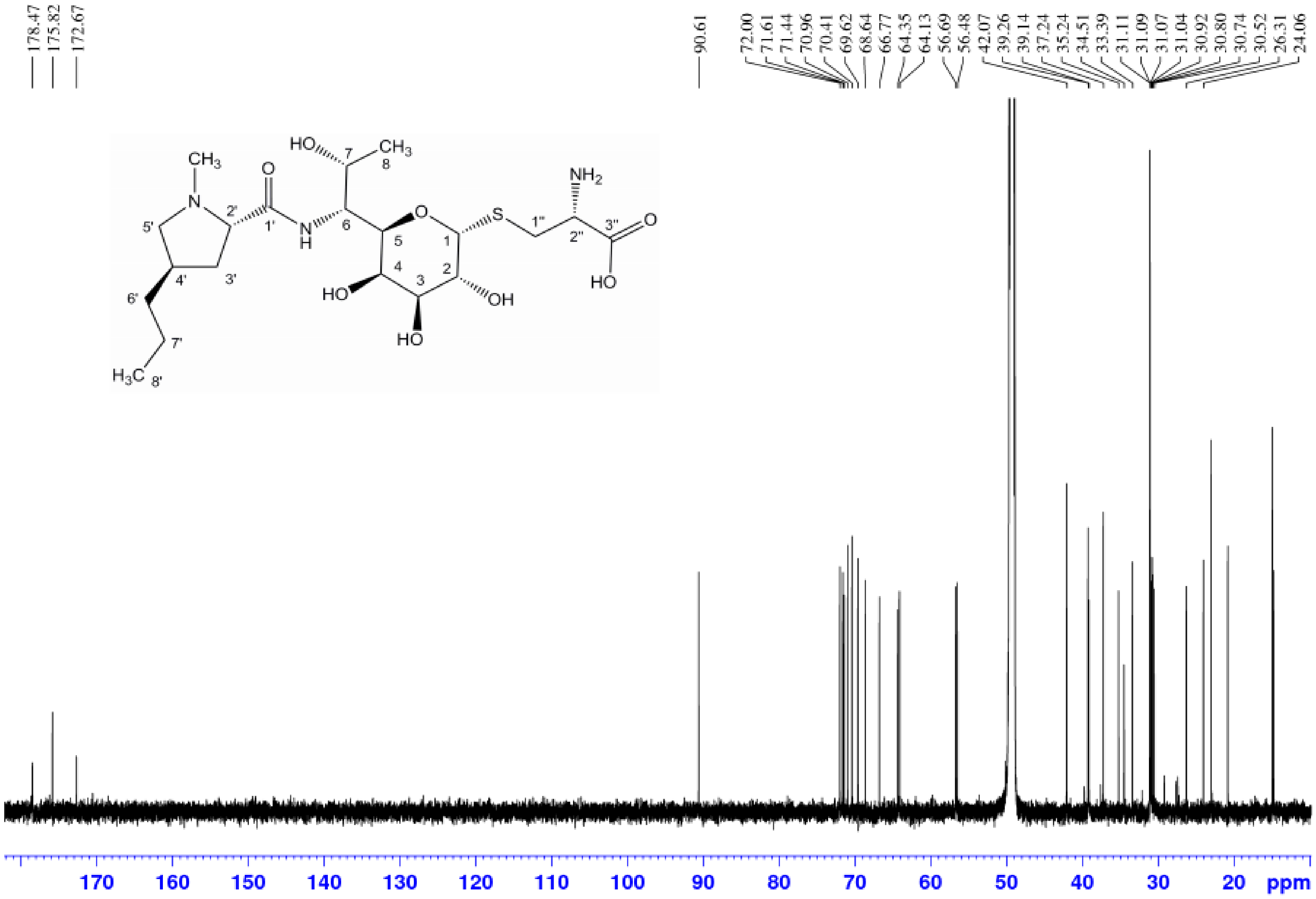

178.47
175.82
172.67
90.61
72.00
71.61
71.44
70.96
70.41
69.62
68.64
66.77
64.35
64.13
56.69
56.48
42.07
39.26
39.14
37.24
35.24
34.51
33.39
31.11
31.09
31.07
31.04
30.92
30.80
30.74
30.52
26.31
24.06
170
160
150
140
130
120
110
100
90
80
70
60
50
40
30
20
ppm

**E.**

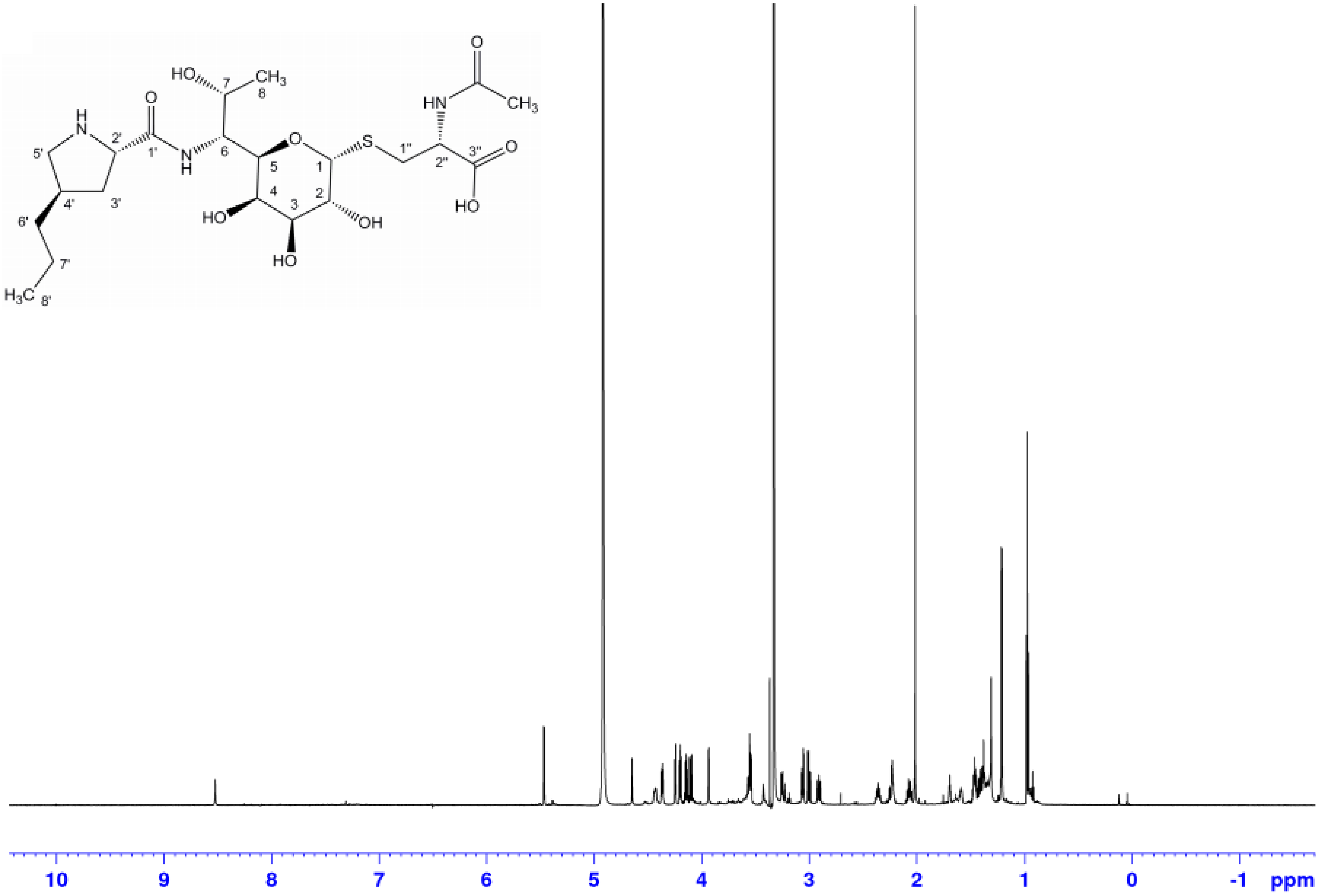
HO
7
CH3
8
O
HN
CH3
O
H
N
2'
5'
1'
N
H
6
5
O
1
S
1"
2"
3"
O
HO
4'
3'
4
3
2
HO
OH
HO
6'
7'
H3C
8'
10
9
8
7
6
5
4
3
2
1
0
-1
ppm

F.

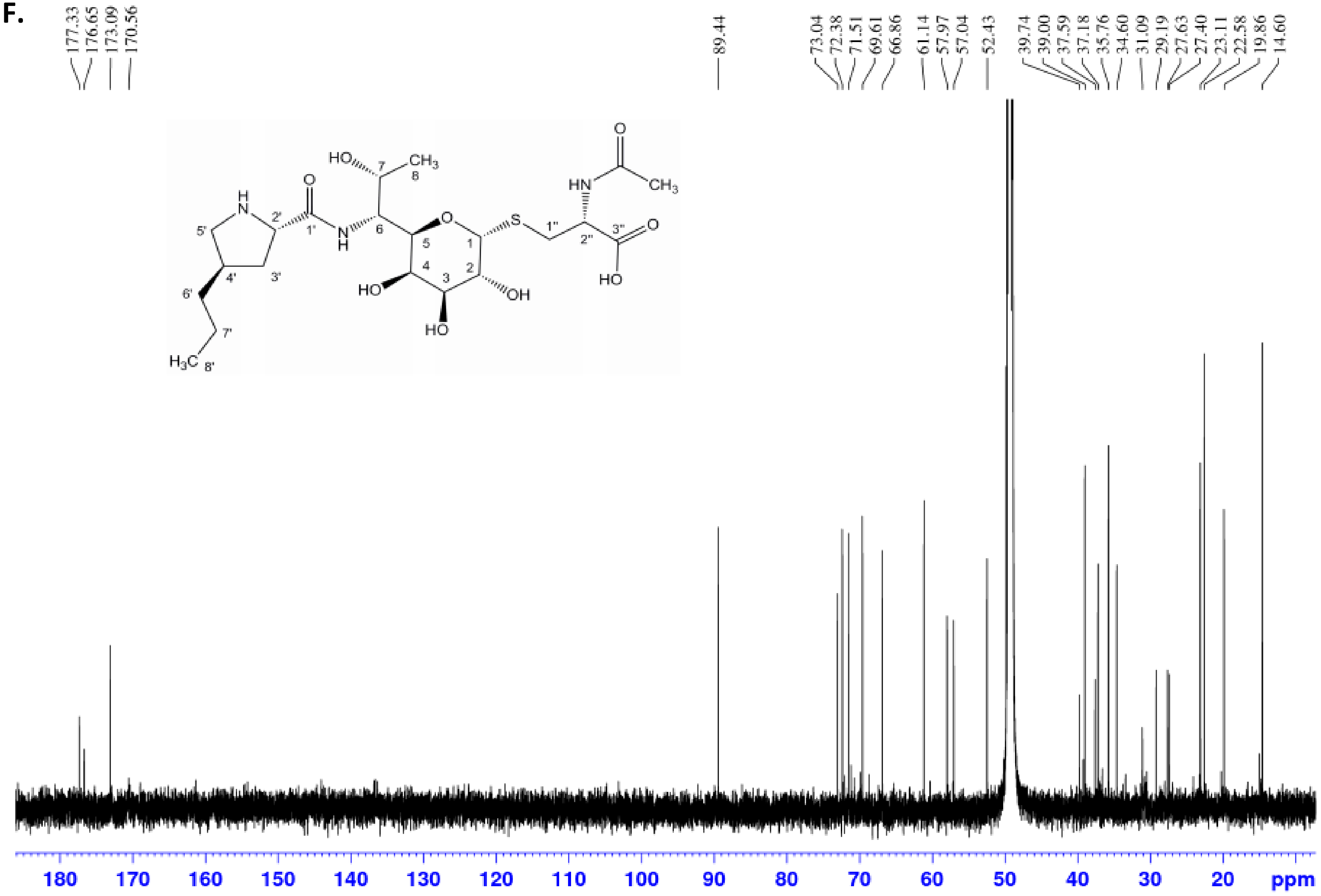
177.33
176.65
173.09
170.56
89.44
73.04
72.38
71.51
69.61
66.86
61.14
57.97
57.04
52.43
39.74
39.00
37.59
37.18
35.76
34.60
31.09
29.19
27.63
27.40
23.11
22.58
19.86
14.60
HO
7
CH3
8
O
HN
CH3
H
N
2'
1'
N
H
6
5
O
1
S
1''
2''
3''
O
HO
5'
4'
3'
6'
7'
H3C
8'
4
3
2
HO
HO
OH
180
170
160
150
140
130
120
110
100
90
80
70
60
50
40
30
20
ppm

G.

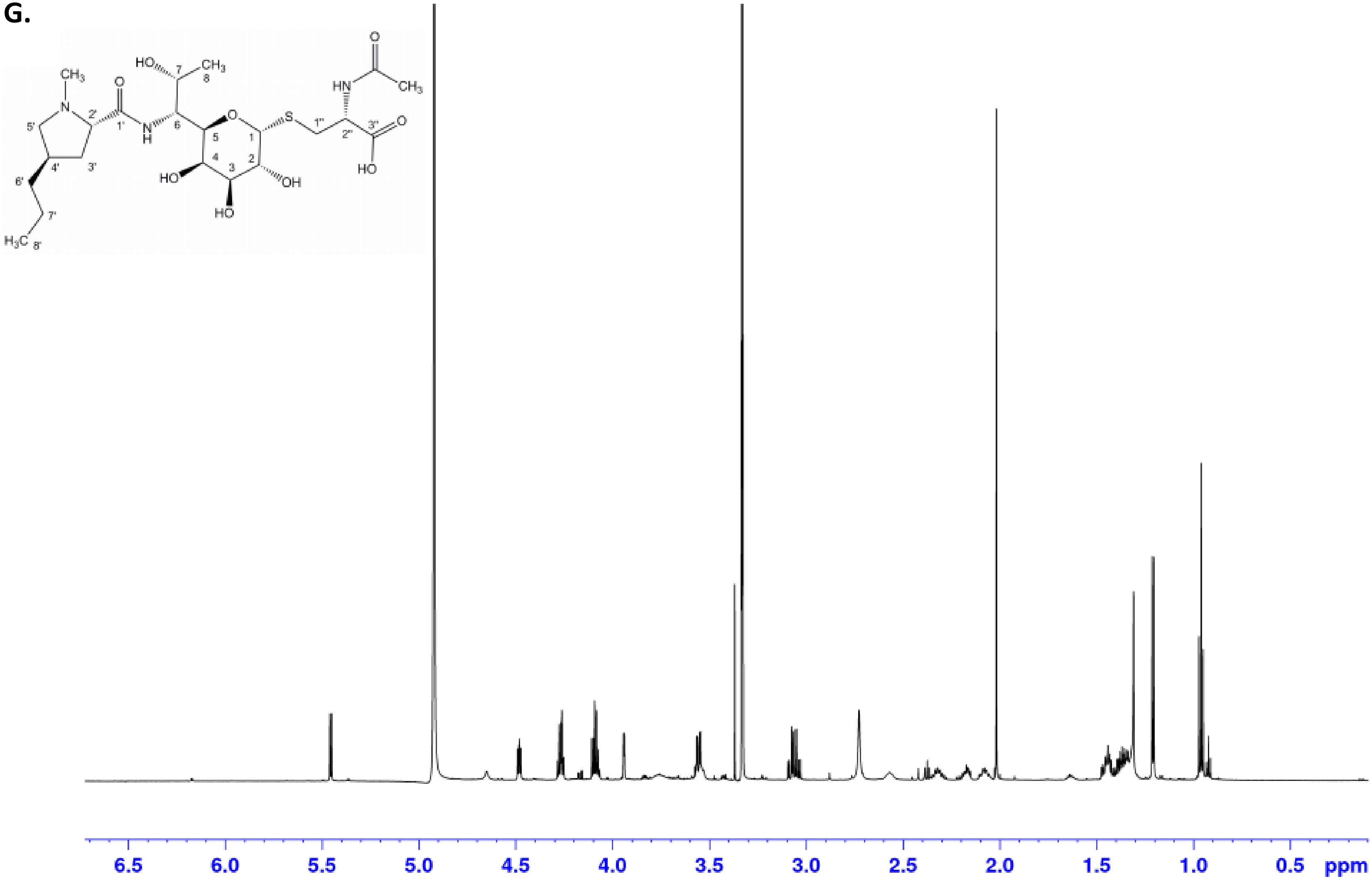

**H.**

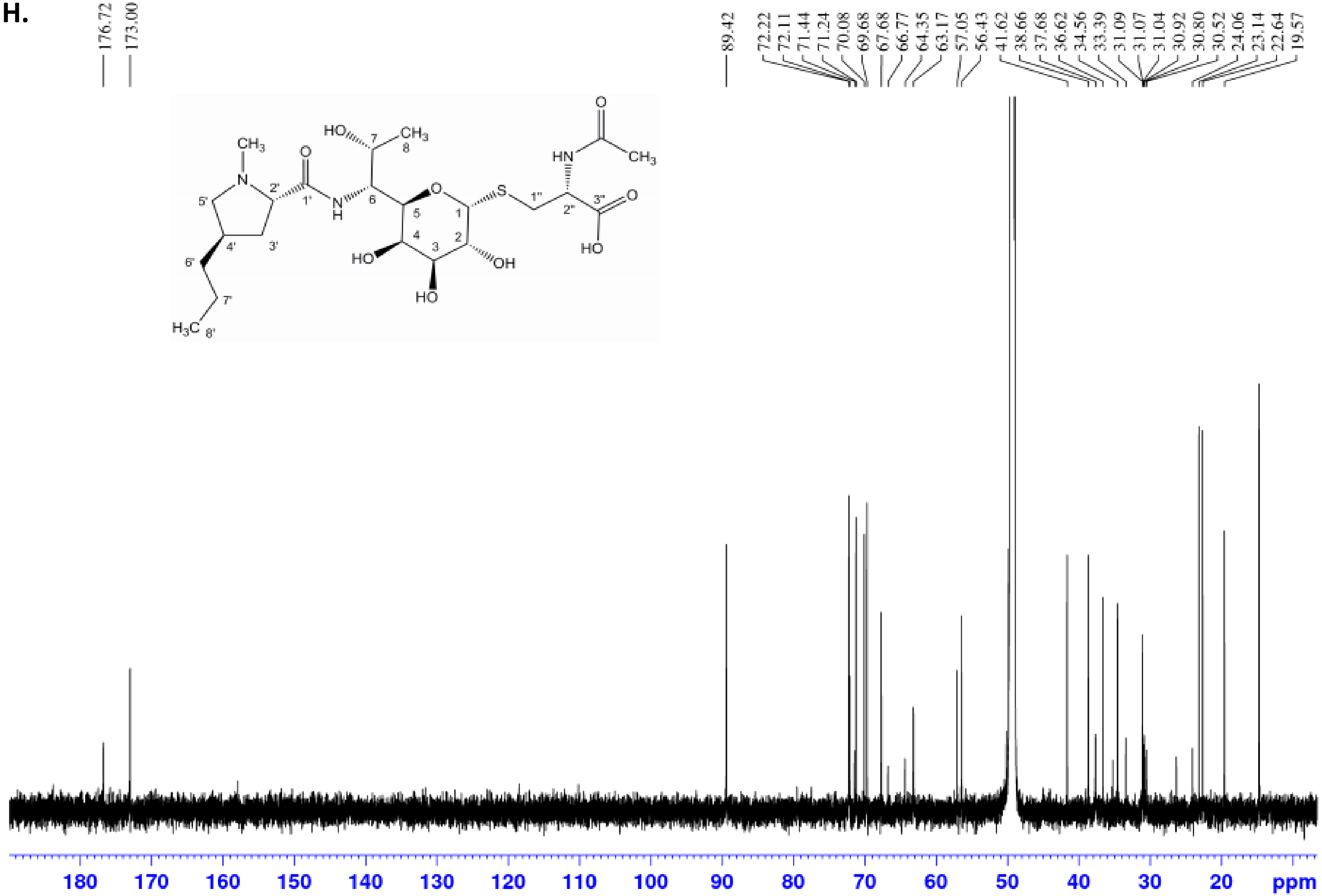

176.72
173.00
89.42
72.22
72.11
71.44
71.24
70.08
69.68
67.68
66.77
64.35
63.17
57.05
56.43
41.62
38.66
37.68
36.62
34.56
33.39
31.09
31.07
31.04
30.92
30.80
30.52
24.06
23.14
22.64
19.57
180
170
160
150
140
130
120
110
100
90
80
70
60
50
40
30
20
ppm

**Fig. S2** NMR spectra of lincomycin intermediates **465**, **479**, **507**, and **521** (700.13 MHz for $^{1}H$, 176.05 MHz for $^{13}C$, $CD_3OD$, 20 °C): A. $^{1}H$ NMR spectrum of compound **465**; B. $^{13}C$ NMR spectrum of compound **465**; C. $^{1}H$ NMR spectrum of compound **479**; D. $^{13}C$ NMR spectrum of compound **479**; E. $^{1}H$ NMR spectrum of compound **507**; F. $^{13}C$ NMR spectrum of compound **507**; G. $^{1}H$ NMR spectrum of compound **521**; H. $^{13}C$ NMR spectrum of compound **521**.

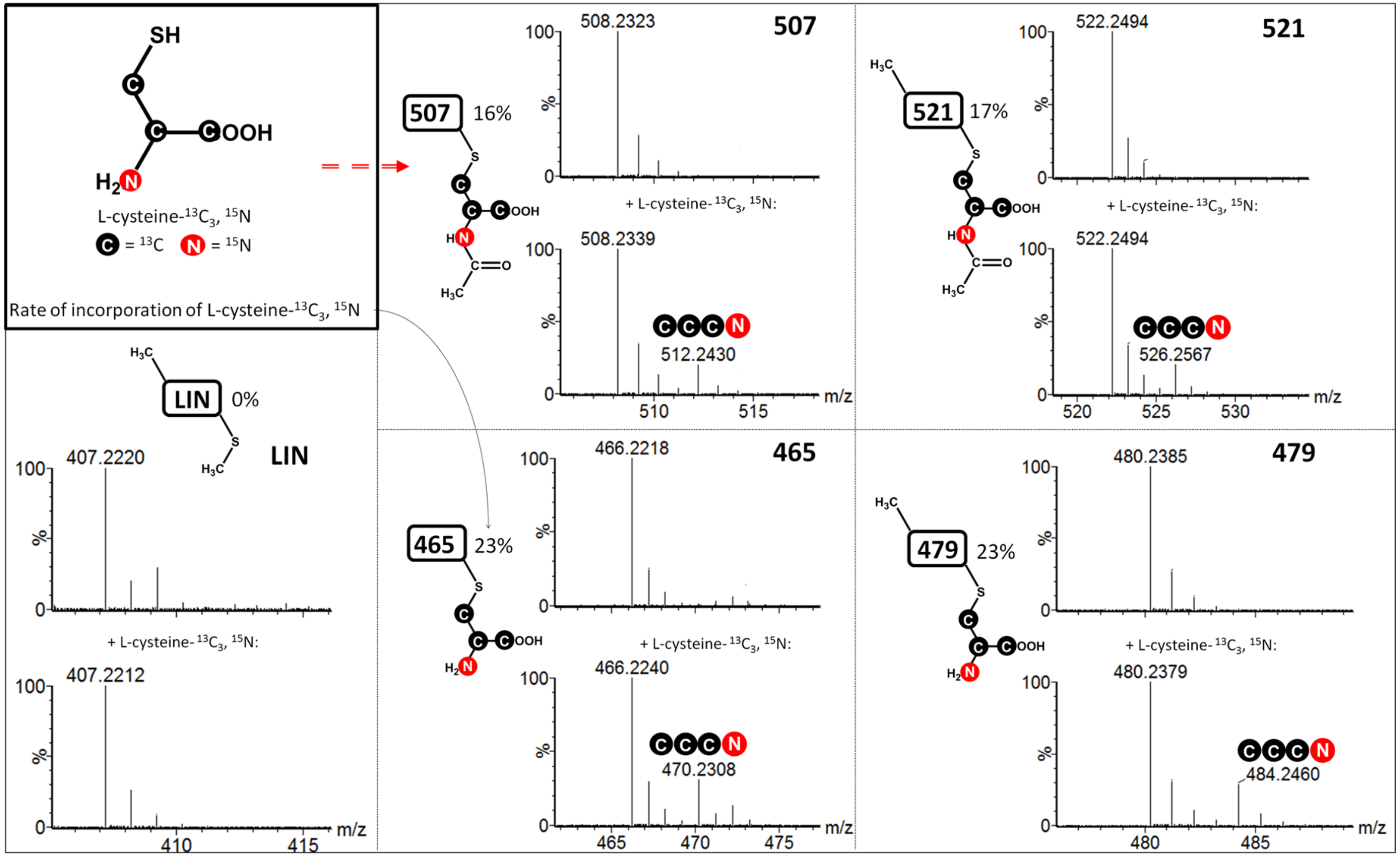


**Fig. S3** Structure elucidation of lincomycin intermediates: Incorporation experiment. MS spectra of lincomycin and its intermediates produced by *Streptomyces lincolnensis* Δ*lmbIH* mutant strain cultured without supplementation (spectra above) and with L-cysteine-$^{13}C_3$,$^{15}N$ supplementation (below) – three $^{13}C$ and a $^{15}N$ atoms were incorporated into the lincomycin intermediates, but not into lincomycin, confirming that cysteine is part of the molecules of the four intermediates.

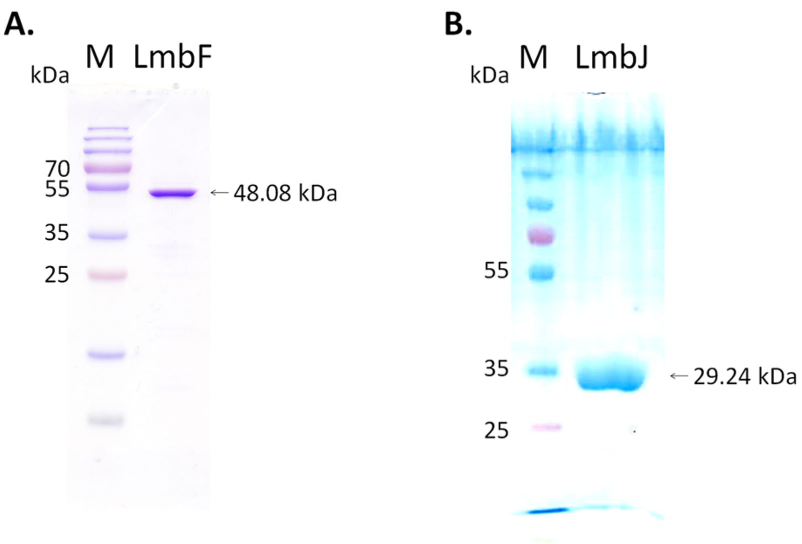


**Fig. S4** SDS PAGE analysis of purified LmbF and LmbJ proteins: A. $His_8$-tagged LmbF – 48.08 kDa; B. $His_6$-tagged LmbJ – 29.24 kDa. M – PageRuler$^{TM}$ prestained protein ladder.

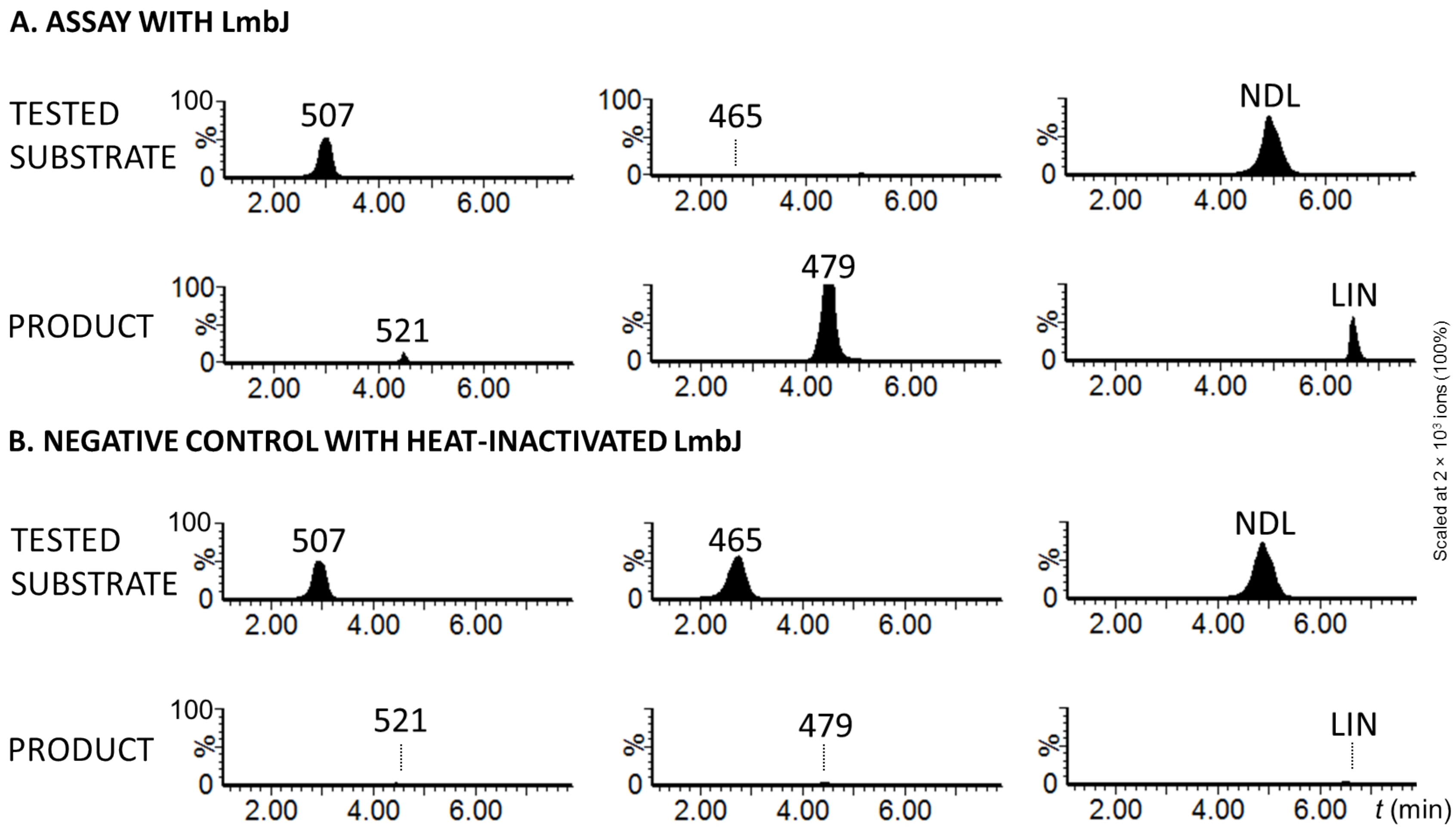


**Fig. S5** Ion-extracted LC-MS chromatograms of *in vitro* assay with **LmbJ**: A. LmbJ; B. heat-inactivated LmbJ – negative control. The reaction products **521** and **479** match retention times, MS spectra and in-source CID MS spectra with compounds **521** and **479** purified from culture broths; the reaction product lincomycin (LIN) matches the same characteristics with authentic standard of LIN, which are depicted in Fig. S1 in ESI.

# 392

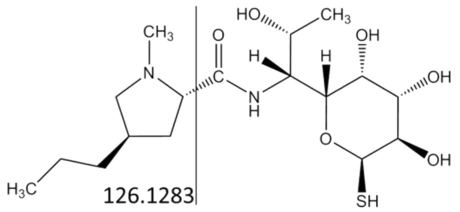


**392**
$[M+H]^+$ 393.2059

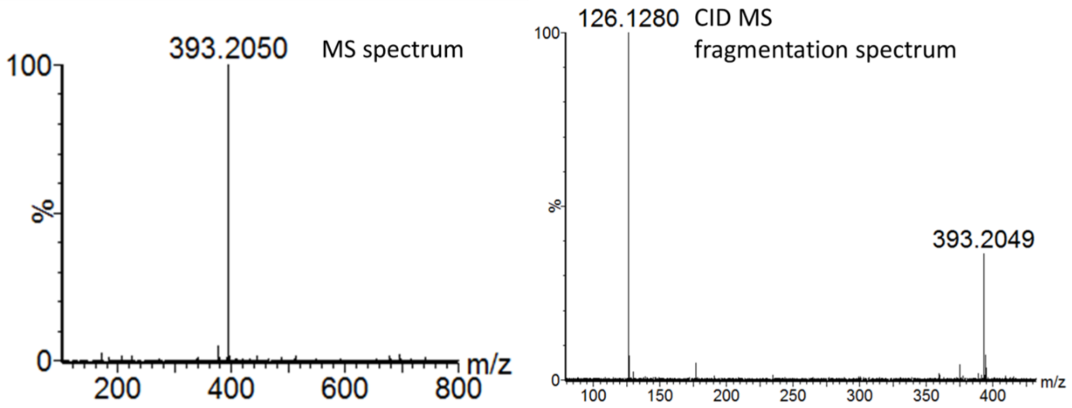


# 378

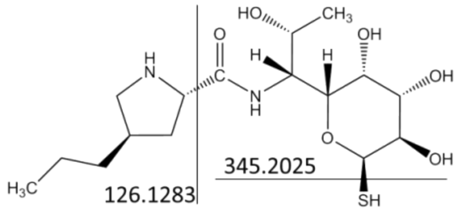


**378**
$[M+H]^+$ 379.1903

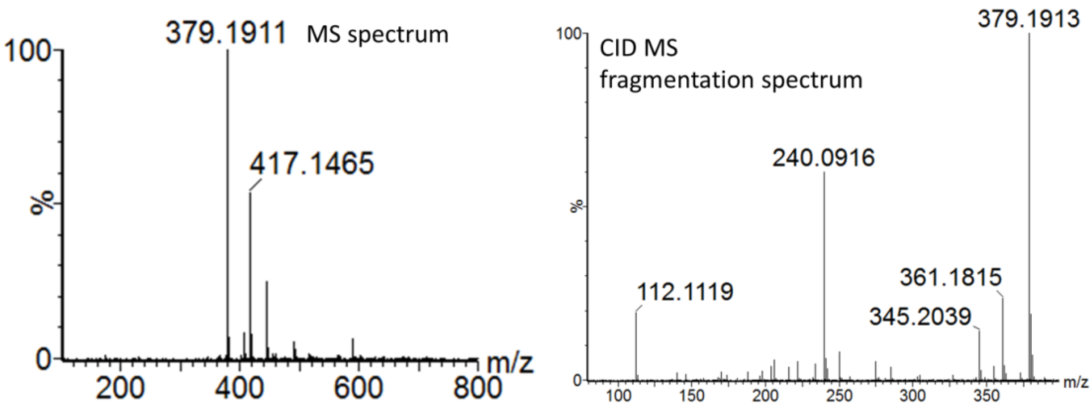


**Fig. S6** Reaction products of LmbF assays (compounds **378** and **392**). MS spectra and in-source CID MS fragmentation spectra of the compounds.

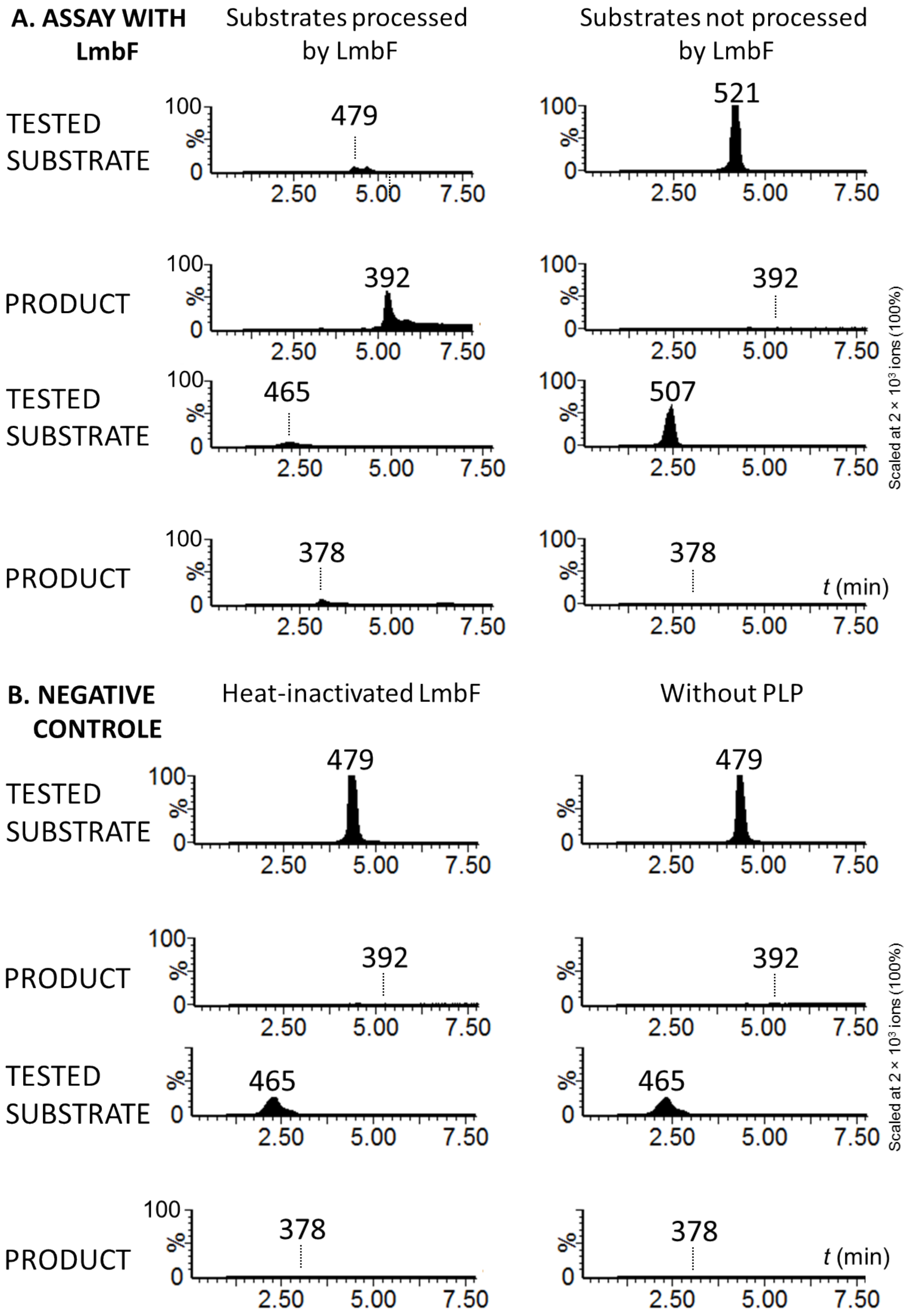


**Fig. S7** Ion-extracted LC-MS chromatograms of *in vitro* assay with LmbF: A. LmbF; B. negative control – heat-inactivated LmbF or absence of PLP.

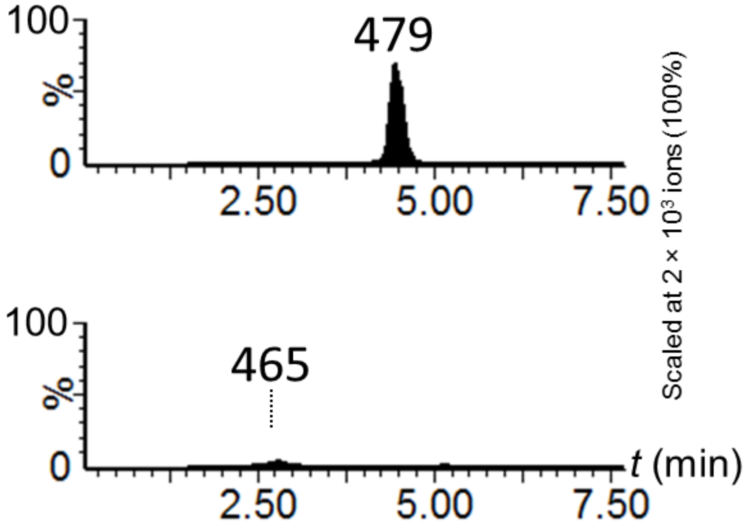


**Fig. S8** Ion-extracted LC-MS chromatograms for culture broth of Δ*lmbF* mutant strain. Comparison with Fig. S7B (ion-extracted LC-MS chromatograms of equimolar amounts of **479** and **465**) shows that Δ*lmbF* mutant strain predominantly produces **479** over **465**.

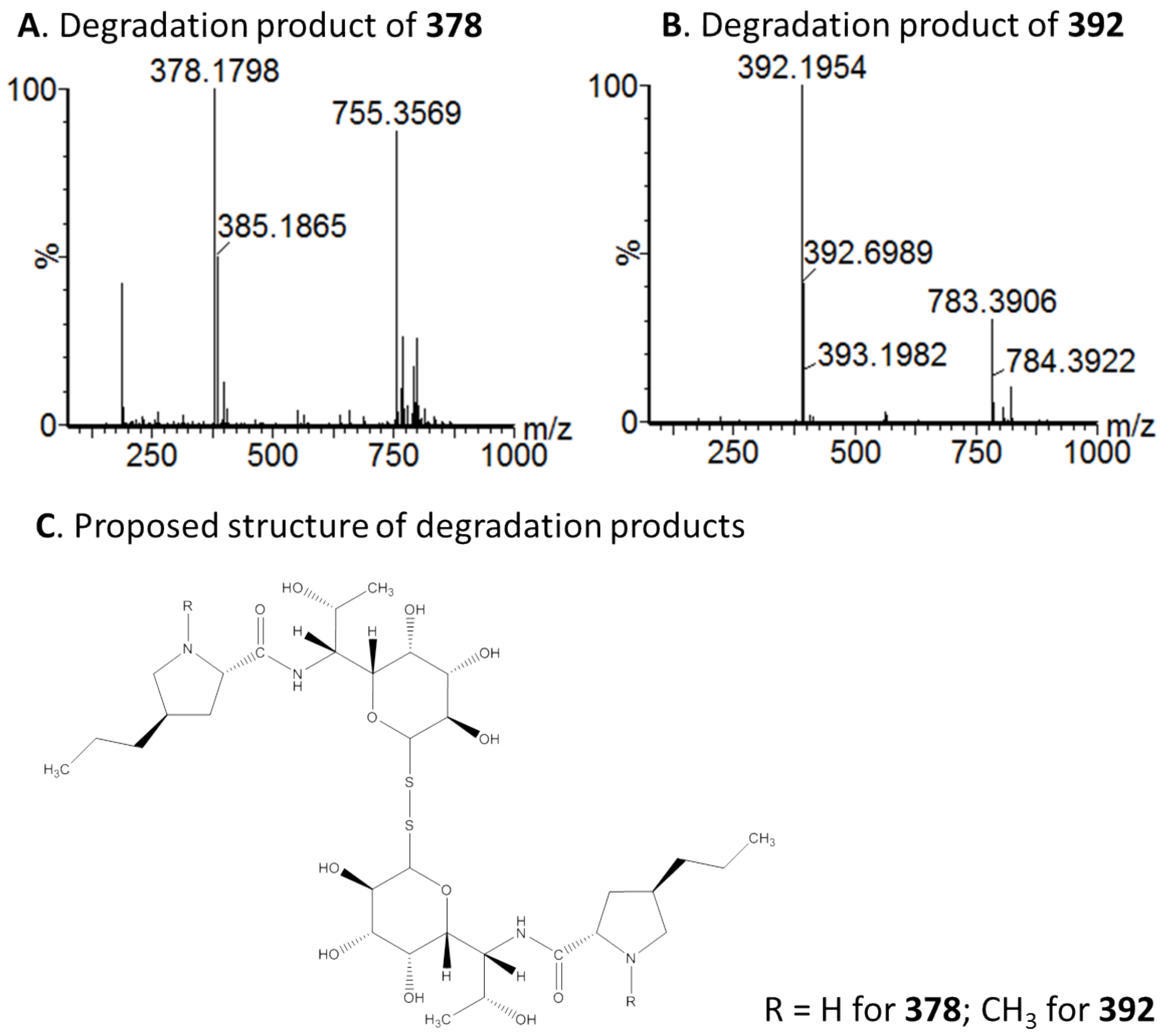


**Fig. S9** MS spectra of degradation products of **378** (A) and **392** (B) and proposed structure (C).

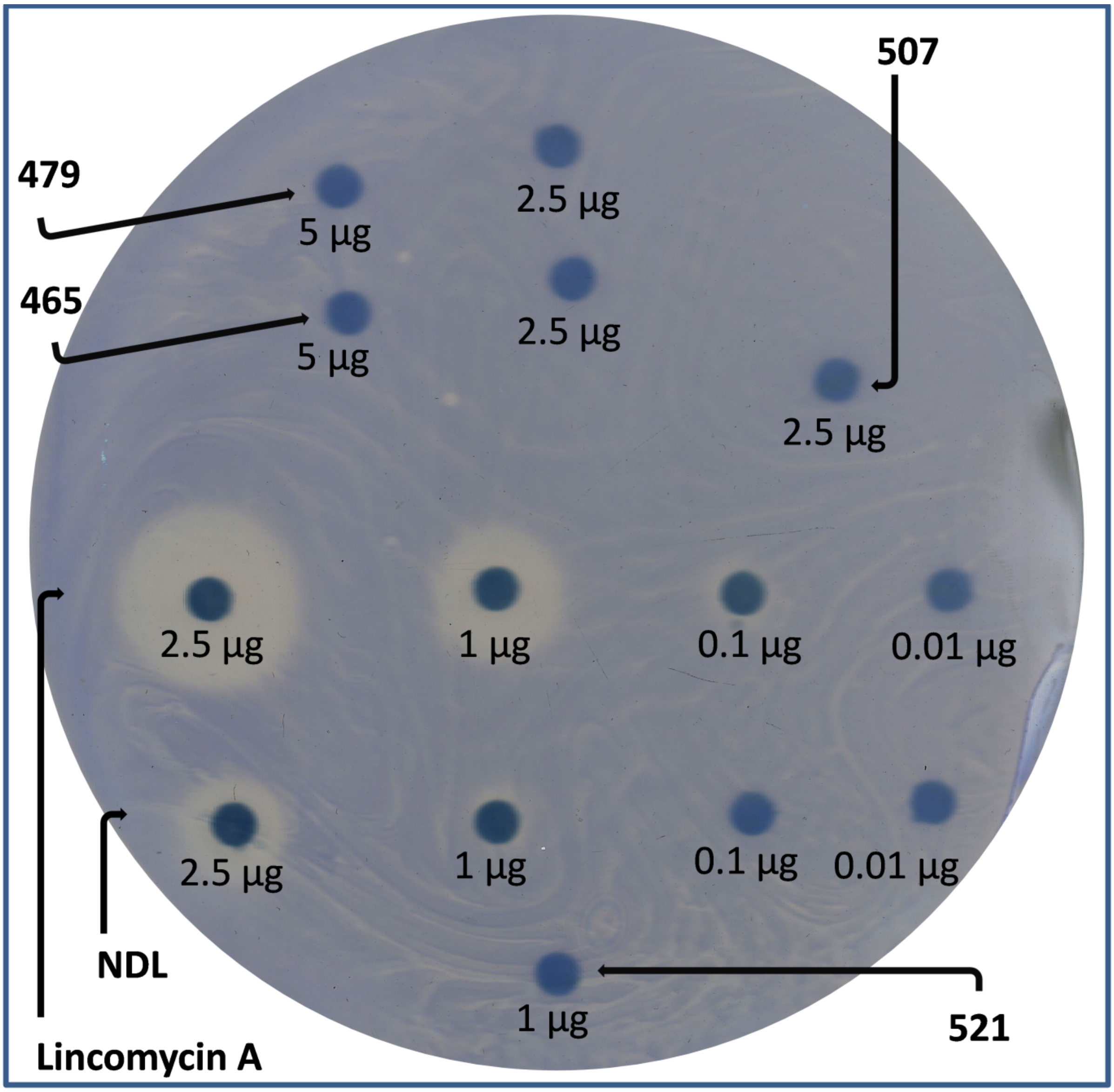


**Fig. S10** Bioassay of antibacterial activity. Testing organism: Gram-positive bacterium *Kocuria rhizophila.* (Colours were inverted, contrast was adjusted).

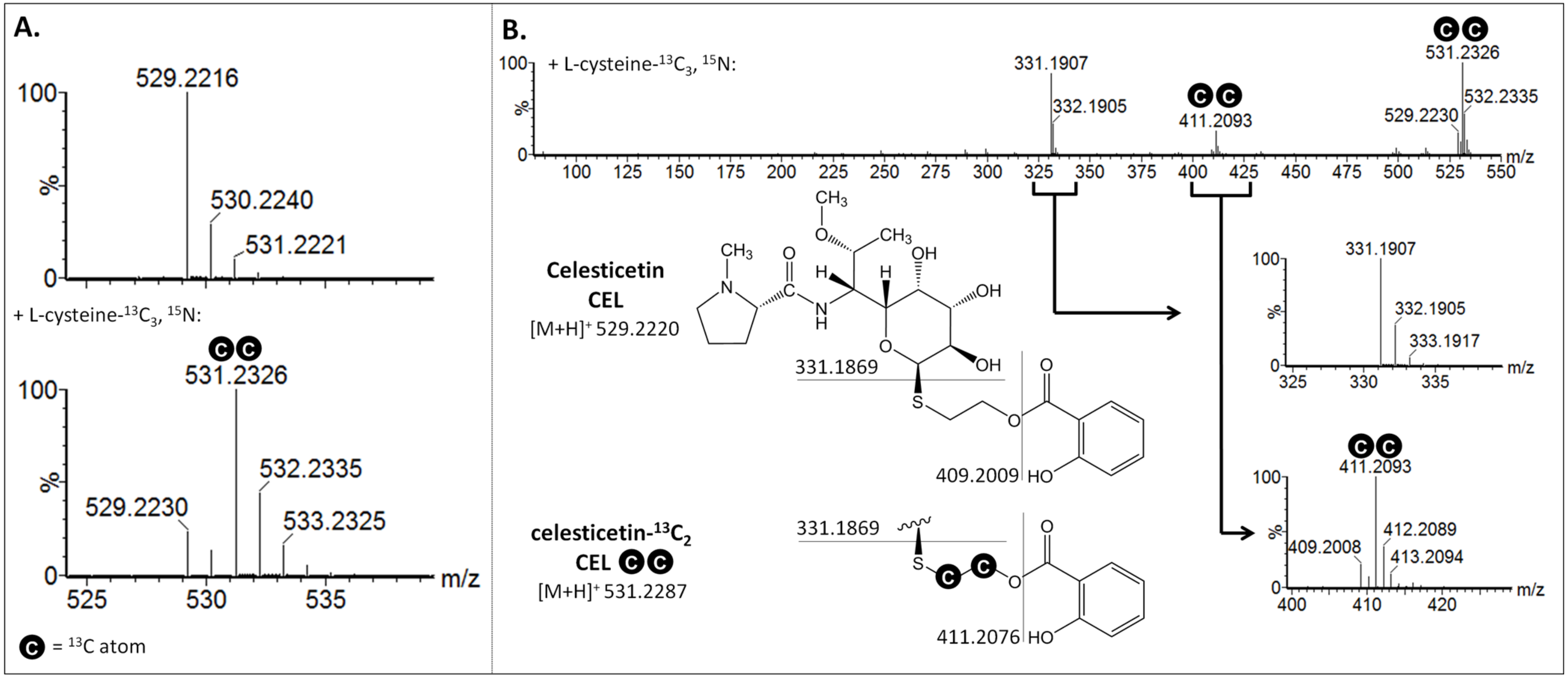


**Fig. S11** Celesticetin biosynthesis: incorporation experiment. A. MS spectrum of celesticetin produced by *Streptomyces caelestis* cultured in GYM medium (above) and in GYM medium supplemented with L-cysteine-$^{13}C_3$,$^{15}N$ (below) – two $^{13}C$ were incorporated into the celesticetin structure; B. In-source CID MS spectrum of celesticetin – fragmentation pattern reveals that the two $^{13}C$ carbons were incorporated into the 2C linker of celesticetin.

## Supplementary references


1. B. Gust, G. L. Challis, K. Fowler, T. Kieser and K. F. Chater, *Proceedings of the National Academy of Sciences of the United States of America*, 2003, **100**, 1541.
2. M. Koberska, J. Kopecky, J. Olsovska, M. Jelinkova, D. Ulanova, P. Man, M. Flieger and J. Janata, *Folia Microbiologica*, 2008, **53**, 395.
3. J. Janata, S. Kadlcik, M. Koberska, D. Ulanova, Z. Kamenik, P. Novak, J. Kopecky, J. Novotna, B. Radojevic, K. Plhackova, R. Gazak and L. Najmanova, *Plos One*, 2015, **10**.
4. Q. F. Zhao, M. Wang, D. X. Xu, Q. L. Zhang and W. Liu, *Nature*, 2015, **518**, 115.
5. H. J. Hong, M. I. Hutchings, L. M. Hill and M. J. Buttner, *Journal of Biological Chemistry*, 2005, **280**, 13055.
6. T. Kieser, M. Bibb, M. J. Buttner, K. F. Chater and D. Hopwood, *Practical Streptomyces Genetics*, The John Innes Foundation, Norwich, UK, 2000.
7. L. Najmanova, D. Ulanova, M. Jelinkova, Z. Kamenik, E. Kettnerova, M. Koberska, R. Gazak, B. Radojevic and J. Janata, *Folia Microbiologica*, 2014, **59**, 543.
8. R. M. Atlas, CRC press, edited by R. M. Atlas, 2010.
9. Z. Kamenik, J. Kopecky, M. Mareckova, D. Ulanova, J. Novotna, S. Pospisil and J. Olsovska, *Analytical and Bioanalytical Chemistry*, 2009, **393**, 1779.
10. J. Olsovska, M. Jelinkova, P. Man, M. Koberska, J. Janata and M. Flieger, *Journal of Chromatography A*, 2007, **1139**, 214.
11. S. Kadlcik, T. Kucera, D. Chalupska, R. Gazak, M. Koberska, D. Ulanova, J. Kopecky, E. Kutejova, L. Najmanova and J. Janata, *Plos One*, 2013, **8**.
12. L. Najmanova, E. Kutejova, J. Kadlec, M. Polan, J. Olsovska, O. Benada, J. Novotna, Z. Kamenik, P. Halada, J. Bauer and J. Janata, *Chembiochem*, 2013, **14**, 2259.
13. S. F. Altschul, T. L. Madden, A. A. Schäffer, J. Zhang, Z. Zhang, W. Miller and D. J. Lipman, *Nucleic Acids Research,* 1997, **25**, 3389.
14. A. Marchler-Bauer, S. N. Lu, J. B. Anderson, F. Chitsaz, M. K. Derbyshire, C. DeWeese-Scott, J. H. Fong, L. Y. Geer, R. C. Geer, N. R. Gonzales, M. Gwadz, D. I. Hurwitz, J. D. Jackson, Z. X. Ke, C. J. Lanczycki, F. Lu, G. H. Marchler, M. Mullokandov, M. V. Omelchenko, C. L. Robertson, J. S. Song, N. Thanki, R. A. Yamashita, D. C. Zhang, N. G. Zhang, C. J. Zheng and S. H. Bryant, *Nucleic Acids Research*, 2011, **39**, D225.
15. D. Ulanova, J. Novotna, Y. Smutna, Z. Kamenik, R. Gazak, M. Sulc, P. Sedmera, S. Kadlcik, K. Plhackova and J. Janata, *Antimicrobial Agents and Chemotherapy*, 2010, **54**, 927.
